# Improvement of different vaccine delivery systems for cancer therapy

**DOI:** 10.1186/1476-4598-10-3

**Published:** 2011-01-07

**Authors:** Azam Bolhassani, Shima Safaiyan, Sima Rafati

**Affiliations:** 1 Molecular Immunology and Vaccine Research Laboratory, Pasteur Institute of Iran, Tehran, Iran

## Abstract

Cancer vaccines are the promising tools in the hands of the clinical oncologist. Many tumor-associated antigens are excellent targets for immune therapy and vaccine design. Optimally designed cancer vaccines should combine the best tumor antigens with the most effective immunotherapy agents and/or delivery strategies to achieve positive clinical results. Various vaccine delivery systems such as different routes of immunization and physical/chemical delivery methods have been used in cancer therapy with the goal to induce immunity against tumor-associated antigens. Two basic delivery approaches including physical delivery to achieve higher levels of antigen production and formulation with microparticles to target antigen-presenting cells (APCs) have demonstrated to be effective in animal models. New developments in vaccine delivery systems will improve the efficiency of clinical trials in the near future. Among them, nanoparticles (NPs) such as dendrimers, polymeric NPs, metallic NPs, magnetic NPs and quantum dots have emerged as effective vaccine adjuvants for infectious diseases and cancer therapy. Furthermore, cell-penetrating peptides (CPP) have been known as attractive carrier having applications in drug delivery, gene transfer and DNA vaccination. This review will focus on the utilization of different vaccine delivery systems for prevention or treatment of cancer. We will discuss their clinical applications and the future prospects for cancer vaccine development.

## Introduction

**Figure 1:**
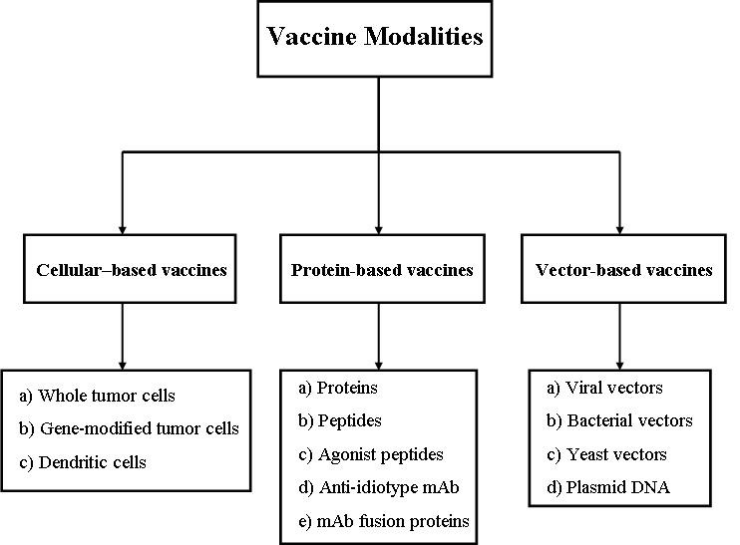
**General vaccine modalities**. Three main vaccination types are totally available against cancer such as cellular-based vaccines, protein-based vaccines and vector-based vaccines. Each these types divide into the subgroups in detail. Among them, DNA vaccines and protein/peptide vaccines have been further involved in vaccine design.

Cancer is a major cause of death in worldwide. Novel diagnostic technologies and screening methods as well as the effective therapeutic agents have diminished mortality for several cancers [1]. The field of vaccinology provides excellent promises to control different infectious and non-infectious diseases. The term of cancer vaccine refers to a vaccine that prevents either infections with cancer-causing viruses or the development of cancer in certain high risk individuals (known as prophylactic cancer vaccine) and treats existing cancer (known as therapeutic cancer vaccine). Generally, several vaccination types are available against different disorders (e.g. cancer). They include recombinant live vector vaccines (viral and/or bacterial vector vaccines), nucleic acid vaccines (DNA and/or RNA replicon vaccines), protein and peptide vaccines, viral-like particles (VLP) vaccines, whole cell vaccines (dendritic cell-based and tumor cell-based vaccines), edible vaccines and combined approaches (e.g. prime-boost vaccination) [2,3]. Figure 1 shows the general vaccine modalities.

The presence of antigens on the surface of tumor cells recognized by cytotoxic and T-helper lymphocytes is essential for effective immune responses and for the development of specific cancer vaccines. In order to augment the immune response, several strategies have been involved such as a) identification of tumor antigens that should be targeted, b) determination of the desired immune response for optimal vaccine design and c) utilization of efficient vaccine delivery [1,3].

Different studies have identified a large number of cancer-associated antigens, which some are now being used as cancer treatment vaccines both in basic research and clinical trials [4]. Nowadays, an important advance is the development of techniques for identifying antigens that are recognized by tumor-specific T lymphocytes. Tumor antigens have been classified into two broad categories: tumor-specific shared antigens and tumor-specific unique antigens. Shared antigens or tumor-associated antigens (TAAs) are expressed by more than one type of tumor cells. A number of TAA are also expressed on normal tissues, albeit in different amounts [4]. As reported in the official National Cancer Institute website (NCI), representative examples of such shared antigens are the cancer-testis antigens, human epidermal growth factor receptor 2 (HER2/neu protein) and carcinoembryonic antigen (CEA). Unique tumor antigens result from mutations induced through physical or chemical carcinogens; they are therefore expressed only by individual tumors [4]. Tumor-specific unique antigens encompass melanocyte/melanoma differentiation antigens, such as tyrosinase, MART1 and gp100, prostate-specific antigen (PSA) and Idiotype (Id) antibodies. Both tumor-specific shared and unique antigens are applied as a basis for the new cancer vaccines. Optimally designed cancer vaccines should combine the best tumor antigens with the most effective immunotherapy agents and/or delivery strategies to achieve positive clinical results [4]. Therefore, selection of an adequate vaccine-delivery system is fundamental in the design of immune strategies for cancer therapy.

In this review, we discuss the current delivery methods that are assisting in future vaccine success especially DNA-based vaccines. DNA vaccination is a promising approach for inducing both humoral and cellular immune responses. DNA vaccines have emerged as an attractive approach for antigen-specific T cell-mediated immunotherapy to combat cancers. T cell-mediated immunity is critical for cancer immunotherapy and vaccine development. Tumor antigens that are recognized by T cells are likely to be the major inducer of tumor immunity and most promising candidates for tumor vaccines [5]. Clearly, the current approach to immunotherapy mainly relies on the role of CD8+ cytotoxic T lymphocytes (CTL).

Generally, various strategies have been developed to enhance the potency of DNA vaccines such as a) increasing the number of antigen-expressing dendritic cells (DCs) or antigen-loaded DCs, b) improving antigen expression, processing and presentation in DCs and c) enhancing DC and T cell interaction [6,7]. Therefore, at first we will further analyze various DNA delivery systems as a powerful research tool for elucidating effective anti-tumor immune responses. Finally, in this review, we will have a brief overview on delivery of proteins and peptides.

### Enhancement of DNA vaccine potency by different approaches

During the last decade, DNA-based immunization has been promoted as a new approach to prime specific humoral and cellular immune responses to protein antigens [8]. In mouse models, DNA vaccines have been successfully directed against a wide variety of tumors, almost exclusively by driving strong cellular immune responses in an antigen-specific fashion [9]. However, there is still a need to improve the delivery of DNA vaccines and to increase the immunogenicity of antigens expressed from the plasmids [8,9]. For example, tumor burden has been decreased by novel DNA vaccine strategies that deliver cytokines as plasmids directly into tumors in both mouse and human models. Altogether, the selected trials for DNA vaccines have shown that immune responses can be generated in humans, but they also highlight the need for increased potency if this vaccine technology is to be effective [9]. The reasons for the failure of DNA vaccines to induce potent immune responses in humans have not been elucidated. However, it is reasonable to assume that low levels of antigen production, inefficient cellular delivery of DNA plasmids and insufficient stimulation of the innate immune system have roles in low potency of DNA vaccine [10]. Therefore, with further optimization DNA vaccine strategies can be improved, with significant effects on the outcome of immunization. In designing vaccine, clearly regimens, plasmid dose, timing of doses, adjuvants, delivery systems and/or routes of vaccination must be considered [11]. Indeed, efforts to improve these aspects of DNA vaccines have resulted in their enhanced efficacy in animals. However, the uptake of DNA plasmids by cells upon injection is very inefficient. Nowadays, two basic strategies have been applied for increasing DNA vaccine potency including a) physical delivery to achieve higher levels of antigen production and b) formulation with microparticles to target antigen-presenting cells (APCs) [10]. Both approaches are effective in animal models, but have yet to be evaluated fully in human clinical trials.

Generally, the methods of delivering a DNA plasmid are divided into:

I. Physical approaches including:

1. Tattooing

2. Gene gun

3. Ultrasound

4. Electroporation

5. Laser

II. Viral and non-viral delivery systems (Non-physical delivery methods) including:

1. Biological gene delivery systems (viral vectors)

2. Non-biological gene delivery systems (non-viral vectors) such as:

2.1. Cationic lipids/liposomes

2.2. Polysaccharides and cationic polymers

2.3. Micro-/Nano-particles

2.4. Cationic peptides/Cell-penetrating peptides (CPP)

### I. Physical approaches for DNA plasmid delivery

The method of delivering a DNA vaccine can influence the type of immune response induced by the vaccine. Generally, DNA may be administered by different methods such as intradermal (i.d.), intramuscular (i.m.), intranasal (i.n.) and subcutaneous (s.c.) [11]. In many cases, cutaneous administration has been associated with immunological benefits, such as the induction of greater immune responses compared with those elicited by other routes of delivery. However, the results of vaccination via the skin, have sometimes been conflicting, due to the lack of delivery devices that accurately and reproducibly administer vaccines to the skin [12]. In addition, the nasal route as a site of vaccine delivery for both local and systemic effect is currently of considerable interest. The success of intranasally delivered mucosal vaccines has been also limited by lack of effective vaccine formulations or delivery systems suitable for use in humans. Nowadays, the properties of polyacrylate polymer-based particulate systems are studied to facilitate mucosal immune responses [13]. However, conventional vaccinations involve subcutaneous or intradermal inoculations. It has been demonstrated in several preclinical animal models and some clinical studies that intra-tumoral and/or intra-nodal vaccination may be more effective than other routes. In a study reviewed in "*Advances in Cancer Research"*, the sequential use of primary vaccination subcutaneously followed by booster vaccination intra-tumorally produced more effective anti-tumor effects than the use of either route alone [3].

Several factors may influence the route of injection. Recently, the enhanced efficiency is observed by using biolistic techniques, such as the Gene gun or Biojector 2000. It has been reported in mice that approximately 100-fold less DNA is required for a comparable antibody response than what could be achieved with needle injection [11]. Biolistic and needle injections may produce different types of immune responses. In many cases, application of a DNA vaccine by gene gun typically induces T helper type 2 (Th2) reactions whereas needle inoculation triggers a Th1 response. The difference may be due to the use of increased doses for needle injection. It is crucial that this finding is not universal [11]. Some previous studies showed that gold particles used in gene gun bombardment affected the induced-immune response, because gene gun administration using non-coating naked DNA vaccine elicited Th1-bias immune response [14]. Moreover, certain antigens are able to bias the responses irrespective of the route [11].

Several strategies have focused on increasing the number of antigen-expressing dendritic cells (DCs) including intradermal administration through gene gun; intradermal injection followed by laser treatment; intramuscular injection followed by electroporation and intramuscular injection of microencapsulated vaccine.

Some physical delivery technologies for improving gene-based immunization have been listed in number 1 to 5 as following:

#### 1. Tattooing

Tattooing has been recently described as a physical delivery technology for DNA injection to skin cells. This approach, which is similar to the effective smallpox-vaccination technique, seems to decrease the time that is required for the induction of potent immune responses and protective immunity. This effect might be related to the rapid and highly transient nature of antigen production after vaccination. Gene expression after DNA tattooing has been shown to be higher than that after intradermal injection and gene gun delivery [15]. As compared to intramuscular injection, DNA delivery by tattooing seems to produce different gene expression patterns. One study showed that tattooing of 20 μg DNA results at least ten times lower peak values of gene expression than intramuscular injection of 100 μg DNA in mouse model [15]. Gene expression after tattooing showed a peak after six hours that it disappeared over the next four days. On the contrary, the intramuscular injection of DNA resulted in high levels of gene expression with a peak after one week that it was detectable up to one month. Despite the lower dose of DNA and decreased gene expression, DNA delivered by tattoo induced higher antigen-specific cellular as well as humoral immune responses than that by intramuscular DNA injection [15].

Furthermore, the effect of two adjuvants, cardiotoxin and plasmid DNA carrying the mouse granulocyte-macrophage colony-stimulating factor (GM-CSF) has been evaluated on the efficacy of a DNA vaccine delivered either by tattoo or intramuscular needle injection [15]. In this study, a codon modified gene encoding the L1 major capsid protein of the human papillomavirus type 16 (HPV16) was used as a model antigen [15]. The results indicated that molecular adjuvants substantially enhance the efficiency of the HPV16 L1 DNA vaccine when administered intramuscularly. Also, the delivery of the HPV16 L1 DNA in the absence of adjuvants using a tattoo device elicited much stronger and more rapid humoral and cellular immune responses than intramuscular needle delivery together with molecular adjuvants. However, the tattoo delivery of DNA is a cost-effective method that may be used in laboratory conditions when more rapid and more robust immune responses are required [15].

Indeed, the tattoo procedure causes many minor mechanical injuries followed by hemorrhage, necrosis, inflammation, and regeneration of the skin and thus non-specifically stimulates the immune system. Therefore, tattooing may "only" partially substitute for the function of adjuvants [16].

#### 2. Gene gun

The particle-mediated or gene gun technology has been developed as a non-viral method for gene transfer into various mammalian tissues. A broad range of somatic cell types, including primary cultures and established cell lines, has been successfully transfected *ex vivo *or *in vitro *by gene gun technology, either as suspension or adherent cultures [17]. The gene gun is a biolistic device that enables delivered DNA to directly transfect keratinocytes and epidermal Langerhans cells. These events stimulate DC maturation and migration to the local lymphoid tissue, where DCs prime T cells for antigen-specific immune responses [18]. Recently, gene gun-mediated transgene delivery system has been used for skin vaccination against melanoma using tumor-associated antigen (TAA) human gpl00 and reporter gene assays as experimental systems [17].

High expression of epidermal growth factor receptor (EGFR) protein was observed in several types of cancer including breast, bladder, colon and lung carcinomas [14]. In a study in mouse, the immunological and anti-tumor responses was evaluated by administration of the plasmid DNA encoding extracellular domain of human EGFR through three different methods: needle intramuscular administration, gene gun administration using gold-coated DNA and gene gun administration using non-coating DNA [14]. Among these methods, gene gun administration using non-coating plasmid DNA induced the strongest cytotoxic T lymphocyte activity and best anti-tumor effects in lung cancer animal model, which may provide the basis for the design of DNA vaccine in human clinical trial in the future. Altogether, route of DNA immunization and its formulation could represent an important element in the design of EGFR DNA vaccine against EGFR-positive tumor [14]. Furthermore, the effect of the CpG motif was observed to switch the Th2-type cytokine microenvironment produced by gene-gun bombardment in draining lymph nodes. The results showed that the addition of the CpG motif can increase IL-12 mRNA expression in draining lymph nodes whether induced by intradermal injection, intramuscular injection or gene-gun bombardment [19]. These data suggest that delivery of the CpG motif induces a Th1-biased microenvironment in draining lymph nodes. Taken together, the CpG motif can act as a 'danger signal' and an enhancer of Th1 immune response in DNA vaccination [19].

The delivery of HPV DNA vaccines using intradermal administration through gene gun was shown to be the most efficient method of vaccine administration in comparison with routine intramuscular injection. Recently, gene gun has been indicated to be able to deliver non-carrier naked DNA under a low-pressure system [18]. Non-carrier naked therapeutic HPV DNA vaccine significantly resulted in less local skin damage than gold particle-coated DNA vaccination. This approach was also able to enhance HPV antigen-specific T cell immunity and anti-tumor effects as compared to the gold particle-coated therapeutic HPV DNA vaccine [18].

Recently, a HPV16 DNA vaccine encoding a signal sequence linked to an attenuated form of HPV16 E7 (E7 detox) and fused to heat shock protein 70 [(Sig/E7detox/HSP70)] has been used in clinical trials. In a previous study, the immunologic and anti-tumor responses have been evaluated by the pNGVL4a-Sig/E7 (detox)/HSP70 vaccine administered using three different delivery methods including needle intramuscular, biojector and gene gun. According to obtained results, DNA vaccine administered via gene gun generated the highest number of E7-specific CD8+ T cells as compared to needle intramuscular and biojector administrations in mice model [20].

#### 3. Ultrasound

Ultrasound (US) can be used to transiently disrupt cell membranes to enable the incorporation of DNA into cells [21,22]. In addition, the combination of therapeutic US and microbubble echo contrast agents could enhance gene transfection efficiency [23]. In this method, DNA is effectively and directly transferred into the cytosol. This system has been applied to deliver proteins into cells [24], but not yet to deliver antigens into DCs for cancer immunotherapy. *In vitro *and *in vivo *studies have revealed that the technique of ultrasound can aid in the transduction of naked plasmid DNA into colon carcinoma cells. Furthermore, the intra-tumoral injection of naked plasmid DNA followed by ultrasound in a mouse squamous cell carcinoma model resulted in enhanced DNA delivery and gene expression.

Currently, ultrasound has been applied in a clinical trial. A phase II study of repeated intranodal injection of Memgen's cancer vaccine was done using Adenovirus-CD 154 (Ad-ISF35) delivered by ultrasound, in subjects with chronic lymphocytic leukemia/small lymphocytic lymphoma (CLL/SLL) [University of California, San Diego; ID: NCT00849524].

#### 4. Electroporation

Over the past decades, electroporation (EP) technology has remained a reliable laboratory tool for the delivery of nucleic acid molecules into target cells. This approach uses brief electrical pulses that create transient "pores" in the cell membrane, thus allowing large molecules such as DNA or RNA to enter the cell's cytoplasm. Immediately following cessation of the electrical field, these pores would close and the molecules would be trapped in the cytoplasm without causing cell death [25]. Typically, milli- and microsecond pulses have been used for electroporation. Recently, the use of nanosecond electric pulses (10-300 ns) at very high magnitudes (10-300 kV/cm) has been studied for direct DNA transfer to the nucleus *in vitro *[26].

In addition to the increased permeability of target cells, EP may also enhance immune responses through increased protein expression, secretion of inflammatory chemokines and cytokines, and recruitment of antigen-presenting cells (i.e., macrophages, dendritic cells) at the EP site [25]. As a result, both antigen-specific humoral and cellular immune responses are increased by EP-mediated delivery of plasmid DNA in comparison with levels achieved by intramuscular injection of DNA alone. Indeed, the addition of *in vivo *EP has been associated with a consistent enhancement of cell-mediated and humoral immune responses in small and large animals, supporting its use in humans [25,27]. Subsequently, a comparison of ultrasound versus electroporation (EP) demonstrated that EP can significantly enhance the transfection efficiency of naked plasmid DNA into skeletal muscle against ultrasound [1].

Recently, EP-mediated delivery of plasmid DNA has been shown to be effective as a boosting vaccine in mice primed with DNA alone, possibly owing to the high level of antigen production obtained by the EP-booster vaccine. Interestingly, this regimen was more effective than the one consisting of two doses of DNA with EP [10]. Actually, this approach might be very attractive because it would eliminate the need for two different types of vaccine. For example, the use of a DNA vaccine expressing the CTL epitope AH1 from colon carcinoma CT26 indicated that effective priming and tumor protection in mice are highly dependent on vaccine dose and volume [28]. Indeed, electroporation during priming with the optimal vaccination protocol did not improve AH1-specific CD8+ T cell responses. In contrast, electroporation during boosting strikingly improved vaccine efficiency. Consequently, prime/boost with naked DNA followed by electroporation dramatically increased T-cell mediated immunity as well as antibody response [28]. Further work will be required to determine the mode of action of this prime-boost approach.

An electroporation driven DNA vaccination strategy has been investigated in animal models for treatment of prostate cancer. Plasmid expressing human PSA gene (phPSA) was delivered *in vivo *by intra-muscular electroporation, to induce effective anti-tumor immune responses against prostate antigen expressing tumors [29]. The results showed that the phPSA vaccine therapy significantly delayed the appearance of tumors and resulted in prolonged survival of the animals. Four-dose vaccination regimen resulted in a significant production of IFN-γ and provided optimal immunological effects in immunized animals. Moreover, co-administration of the synthetic CpG with phPSA increased anti-tumor responses, preventing tumor occurrence in 54% of treated animals [29]. Therefore, *in vivo *electroporation mediated vaccination is a safe and effective modality for the treatment of prostate cancer and has a potential to be used as an adjuvant therapy.

The researchers have used HPV E6 and E7 tumor antigens to generate an optimal HPV DNA vaccine by codon optimization (Co), fusion of E6 and E7 (E67), addition of a tissue plasminogen activator (tpa) signal sequence, addition of CD40 ligand (CD40L) or Fms-like tyrosine kinase-3 ligand (Flt3L). When E6 (Co) and E7 (Co) were fused (E67 (Co)), E6/E7 antigen-specific CD8 (+) T cell responses decreased, but the preventive anti-tumor effect was rather improved. Interestingly, Flt3L-fused HPV DNA vaccine exhibited stronger E6- and E7-specific CD8+ T cell responses as well as therapeutic anti-tumor effects than that of CD40L linked HPV DNA vaccine [30]. Finally, the optimal construct, tFE67(Co), was generated by using tpa signal sequence, Flt3L, fusion of E6 and E7 and codon optimization, which induced 23 and 25 times stronger E6- and E7-specific CD8+ T cell responses than those of initial E67 fusion construct. It is noteworthy that inclusion of electroporation in intramuscular immunization of tFE67 (Co) further increased HPV-specific CD8+ T cell responses, leading to complete tumor regression in a therapeutic vaccination [30]. This vaccine regimen induced 34- and 49-fold higher E6- and E7-specific CD8+ T cell response, respectively, as compared to responses observed following vaccination with E67. Thus, these evidences suggest that tFE67 (Co) delivered with electroporation is a promising therapeutic HPV DNA vaccine against cervical cancer [30].

It is critical that intracellular targeting of tumor antigens through its linkage to immunostimulatory molecules such as calreticulin (CRT) can improve antigen processing and presentation through the MHC class I pathway and increase cytotoxic CD8+ T cell production. However, even with these enhancements, the efficacy of such immunotherapeutic strategies is dependent on the identification of an effective method of DNA administration [31]. A comparison was performed between three vaccination methods including conventional intramuscular injection, electroporation-mediated intramuscular delivery and epidermal gene gun-mediated particle delivery using the pNGVL4a-CRT/E7 (detox) DNA vaccine. This study showed that vaccination via electroporation generated the highest number of E7-specific cytotoxic CD8+ T cells, which correlated to improved outcomes in anti-tumor effects [31].

Recently, electroporation has been successfully used to administer several HPV DNA vaccines to mice model as well as rhesus macaques. It has been prompted its use in an ongoing Phase I clinical trial of VGX-3100, a vaccine including plasmids targeting E6 and E7 proteins of both HPV subtypes 16 and 18. The vaccine is proposed to be given to patients with a history of CIN 2 and 3 that have been treated by surgery [18].

Targeting skin cells in particular by Cyto Pulse is more effective than other available intramuscular electroporation systems. Two clinical vaccine delivery systems have been designed by Cyto Pulse including DermaVax™ and Easy Vax™. Easy Vax™ primarily targets the epidermis layer of skin as used in mass-scale prophylactic virus vaccination. In contrast, Derma Vax™ primarily targets the dermis layer of skin. This system is suitable for when high doses and robust immune responses are desired such as cancer vaccines and gene therapy. Clinical trials in progress and planned using Derma Vax include 1) Prostate cancer (Phase I/II), start: December 2008, Uppsala University Hospital and Department of Oncology and Pathology, Karolinska Institute; 2) Colorectal cancer (Phase I/II), start: October 2009, Department of Oncology and Pathology, Karolinska Hospital and The Swedish Institute for Infectious Disease Control, Karolinska Institute. In this study, DNA vaccine was delivered by intradermal electroporation to treat colorectal cancer (El-porCEA; ID: NCT01064375). The purpose of this study was to evaluate the safety and immunogenicity of a CEA DNA immunization approach in patients with colorectal cancer.

Hepatitis C virus DNA vaccine showed acceptable safety when delivered by Inovio Biomedical's electroporation delivery system in phase I/II clinical study at Karolinska University Hospital. ChronVac-C is a therapeutic DNA vaccine being given to individuals already infected with hepatitis C virus with the aim to clear the infection by boosting a cell-mediated immune response against the virus. This clinical study is being conducted at the Infectious Disease Clinic and Center for Gastroenterology at the Karolinska University Hospital in Sweden. This vaccination was among the first infectious disease DNA vaccine to be delivered in humans using electroporation-based DNA delivery.

A phase I dose escalation trial of plasmid interleukin (IL)-12 electroporation was carried out in patients with metastatic melanoma. This report described the first human trial, of gene transfer utilizing *in vivo *DNA electroporation. The results indicated that the modality was safe, effective, reproducible and titratable [32].

Altogether, the electroporation with DNA vaccines has been investigated in several clinical trials for cancer therapy. They include: a) Intratumoral IL-12 DNA plasmid (pDNA) [ID: NCT00323206, phase I clinical trials in patients with malignant melanoma]; 2) Intratumoral VCL-IM01 (encoding IL-2) [ID: NCT00223899; phase I clinical trials in patients with metastatic melanoma]; 3) Xenogeneic tyrosinase DNA vaccine [ID: NCT00471133, phase I clinical trials in patients with melanoma]; 4) VGX-3100 [ID: NCT00685412, phase I clinical trials for HPV infections], and 5) IM injection prostate-specific membrane antigen (PSMA)/pDOM fusion gene [ID: UK-112, phase I/II clinical trials for prostate cancer] [1,33].

#### 5. Laser

*In vitro *studies have shown that laser beam can deliver a certain amount of energy (e.g., up to 20 mega electron volts for the first time) onto a target cell, modifying permeability of the cell membrane by a local thermal effect. For therapeutic applications, a further increase in the amount of energy (e.g., up to 250 mega electron volts) is necessary [34]. Recently, this novel technology has been described to be an effective method of enhancing the transfection efficiency of injected plasmids intradermally and inducing antigen-specific CD4+ and CD8+ T cell immune response as well as humoral immunity. This novel technology was only used to show a high potential for therapeutic HPV DNA vaccine development in a limited number of studies [18].

### II. Viral and non-viral delivery systems

Over the past 40 years, DNA delivery has become a powerful research tool for elucidating gene structure, regulation and function. Transfection efficacy is dependent on both the efficiency of DNA delivery into the nucleus and DNA expression, as well [35]. Although a higher expression can usually be achieved with strong promoters and enhancers (e.g., human cytomegalovirus: hCMV) [4,36], improvements in the efficiency of DNA delivery per second have been difficult to achieve. Therefore, most DNA delivery systems operate at three general levels: DNA condensation, endocytosis and nuclear targeting [35].

#### 1. Biological gene delivery systems (viral vectors)

The design of efficient vectors for vaccine development and cancer gene therapy is an area of intensive research. Live vectors (attenuated or non-pathogenic live virus or bacteria) such as *vaccinia *virus and other *poxviruses*, *adenovirus *and BCG have been evolved specifically to deliver DNA into cells and are the most common gene delivery tools used in gene therapy [37,38]. The major advantage of live vectors is that they produce the antigen in its native conformation, which is important for generating neutralizing antibodies and can facilitate antigen entry into the MHC class I processing pathway for the induction of CD8+ CTL [38].

The most effective immunization protocol may involve priming with one type of immunogen and boosting with another. This method may be useful because: 1) one methodology may be more effective in priming naïve cells, while another modality may be more effective in enhancing memory cell function; 2) two different arms of the immune system may be enhanced by using two different modalities (i.e., CD4+ and then CD8+ T cells); and 3) some of the most effective methods of immunization, like the use of recombinant *vaccinia *virus or *adenoviruses*, can be applied for only a limited number of times because of host anti-vector responses. These vectors may be most effective when used as priming agents, followed by boosting with other agents [28].

The very deep knowledge acquired on the genetics and molecular biology of *herpes simplex virus *(HSV) as major human pathogen will surely expand different ideas on the development of potential vectors for several applications to be utilized in human healthcare. These applications include a) delivery of human genes to cells of the nervous system, b) selective destruction of cancer cells, c) prophylaxis against infection with HSV or other infectious diseases and d) targeted infection of specific tissues or organs [39].

Viruses represent ideal nanoparticles due to their regular geometries, well characterized surface properties and nanoscale dimensions. Molecules can be incorporated onto the viral surface with control over their spacing and orientation, and this can be used to add reactivity to specific sites of the capsid [40]. Recombinant *adenoviruses *(Ads) have enormous potential for gene therapy because they are extremely efficient at delivering DNA to target cells, can infect both dividing and quiescent cells, have a large capacity for incorporation of cDNA expression cassettes, and have a low potential for oncogenesis because they do not insert their genome into the host DNA. At present, the engineering of "smart" nanoparticles are based upon recombinant *adenovirus *vectors. Due to the modular nature of the Ad capsid, multiple therapeutic or diagnostic modalities, such as the addition of magnetic resonance imaging contrast agents, radiation sensitizers and antigenic peptides for vaccines, can be incorporated by modifying different sites on the viral capsid [40].

For an ideal vaccine, it is crucial to avoid vector-related immune responses, have relative specificity for transducing DC, and induce high levels of transgene expression. Adenoviral (AdV) vectors can deliver high antigen concentrations, promote effective processing and MHC expression, and stimulate potent cell-mediated immunity. While AdV vectors have performed well in pre-clinical vaccine models, their application to patient care has limitations. Indeed, the *in vivo *administration of AdV vectors is associated with both innate and adaptive host responses that result in tissue inflammation and injury, viral neutralization, and premature clearance of AdV-transduced cells [41]. However, Ads have received extensive clinical evaluation and are used for one-quarter of all gene therapy trials.

In current study, a *retroviral *vector was encapsulated with genetic segment bearing both IL-12 and herpes simplex virus thymidine kinase (HSV-tk) genes [42]. The combined gene delivery resulted in three- to four-fold reduction in tumor size in nude mice bearing xenografted thyroid cancers as compared to single IL-12 gene treatment. However, it is important to consider that multiple gene delivery via *retroviral *vectors is rarely applied due to their limited encapsulation capacity [43]. Moreover, the anti-tumor effects and survival rates in tumor bearing mice were significantly enhanced when IL-2 and IL-12 were delivered simultaneously using a single vaccine viral vector (*Poxvirus*/*vaccinia *viral vector) along with the tumor antigen [44].

Recently, bacteria-based vectors are being investigated as optimal vehicles for antigen and therapeutic gene delivery to tumor cells. Attenuated *Salmonella *strains have shown great potential as live vectors with broad applications in human and veterinary medicine. Only few clinical trials have been conducted so far, and although they have demonstrated the safety of this system, the results on immunogenicity are less than optimal [45]. A convenient DNA vaccine delivery system is oral vaccination using live-attenuated *Salmonella typhimurium*. The use of attenuated *Salmonella *strains as vehicles to deliver plasmid DNA *in vivo *indicated an effective method to induce strong cell-mediated and humoral immune responses at mucosal sites [27].

In clinical studies, a recombinant *vaccinia *virus vector has been developed to express single or multiple T cell co-stimulatory molecules as a vector for local gene therapy in patients with malignant melanoma. This approach generated local and systemic tumor immunity and induced effective clinical responses in patients with metastatic disease [46]. Furthermore, PSA-TRICOM vaccine (prostate-specific antigen plus a TRIad of co-stimulatory molecules; PROSTVAC) includes a priming vaccination with recombinant vaccinia (rV)-PSA-TRICOM and booster vaccinations with recombinant fowlpox (rF)-PSA-TRICOM. Each vaccine consists of the transgenes for PSA, including an agonist epitope, and three immune co-stimulatory molecules (B7.1, ICAM-1, and LFA3; designated TRICOM) [44]. The efficacy of PSA-TRICOM has been evaluated in phase II clinical trials in patients with metastatic hormone-refractory prostate cancer (mHRPC). PANVAC-VF, another poxviral-based vaccine, consists of a priming vaccination with rV encoding CEA (6D), MUC1 (L93), and TRICOM plus booster vaccinations with rF expressing the identical transgenes. CEA (6D) and MUC1 (L93) represent carcinoembryonic antigen and mucin 1 glycoprotein, respectively, with a single amino acid substitution designed to enhance their immunogenicity. This vaccine is currently under evaluation in several different types of CEA or MUC1-expressing carcinomas and in patients with a life expectancy more than three months [47].

**Figure 2:**
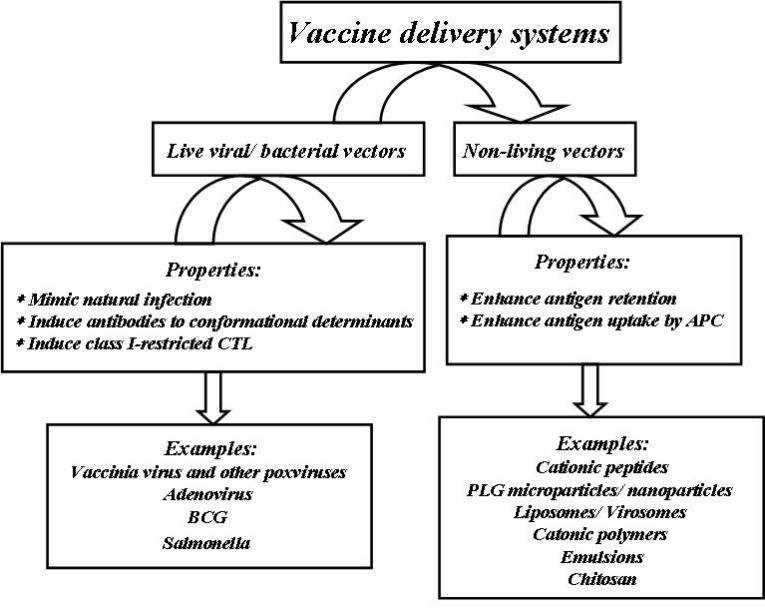
**Live/non-live delivery systems**. Live or biological gene delivery systems include viral and/or bacterial vectors. Non-live or non-biological delivery systems mainly include cationic lipids/liposomes, polysaccharides and cationic polymers, micro-/nano-particles, cationic peptides and cell-penetrating peptides (CPP).

However, there are limitations associated with the use of live viruses or bacteria including their limited DNA carrying capacity, toxicity, immunogenicity, the possibility of random integration of the vector DNA into the host genome and their high cost [48,49]. Non-viral or synthetic vectors have many advantages over their viral counterparts as they are simple, safe and easy to manufacture on a large scale and have flexibility in the size of the transgene to be delivered. Also, these nano-carriers avoid DNA degradation and facilitate targeted delivery to antigen presenting cells [38,50]. Figure 2 generally shows live and non-live delivery systems.

#### 2. Non-biological gene delivery systems (non-viral vectors)

Non-viral vectors must be able to tightly compact and protect DNA, target specific cell-surface receptors, disrupt the endosomal membrane and deliver the DNA cargo to the nucleus [51]. Generally, non-viral vectors include naked DNA, DNA-liposome complexes and DNA-polymer complexes [1,52]. In other way, non-viral particulate vectors used for gene delivery are divided into microspheres, nanospheres and liposomes [53]. The encapsulation of plasmid DNA into micro- or nanospheres can provide protection from the environment prior to delivery and aid in targeting to a specific cell type for efficient delivery [1]. Liposomes and polymers have also been utilized for the delivery of plasmid DNA, although they exhibit some toxicity *in vivo*. The association of DNA with lipids or polymers results in positively charged particles small enough for cell entry through receptor-mediated endocytosis. One example of the utilization of liposomes is the intravenous delivery of the survivin promoter as a DNA-liposome complex which has been shown to be highly specific and has the ability to suppress cancer growth *in vitro *and *in vivo *[1]. The injection of DNA complexed to oxidized or reduced mannan-poly-L-lysin *in vivo *resulted in the production of antibodies with anti-tumor potential as compared to DNA alone in mice model. Formulation of plasmid DNA with a non-ionic block copolymer, poloxamer CRL1005, and the cationic surfactant benzalkonium chloride resulted in a stable complex that elicited the efficient antigen-specific cellular and humoral immune responses and is currently being evaluated in a Phase II clinical trial for melanoma [1].

##### 2.1. Cationic lipids/liposomes

**Figure 3:**
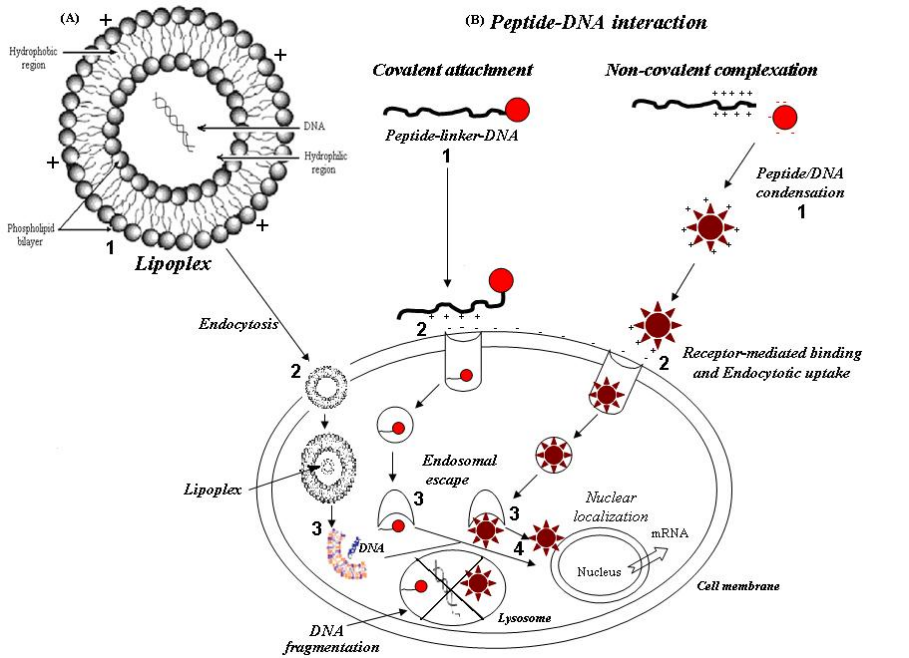
**A) Lipoplex-mediated transfection:**1) Cationic lipids forming micellar structures called liposomes are complexed with DNA to create lipoplexes2) The complexes are internalized by endocytosis, resulting in the formation of a double-layer inverted micellar vesicle. 3) During the maturation of the endosome into a lysosome, the endosomal wall might rupture, releasing the contained DNA into the cytoplasm and potentially towards the nucleus. 4) DNA imported into the nucleus might result in gene expression. Alternatively, DNA might be degraded within the lysosome. **B) peptide-based nucleic acid delivery systems**: Both covalent attachment and/or non-covalent complexes of peptide-DNA are acting similar to lipid-based systems. The designed cationic peptides must be able to 1) tightly condense DNA into small, compact particles; 2) target the condensate to specific cell surface receptors; 3) induce endosomal escape; and 4) target the DNA cargo to the nucleus for reporter gene expression.

Lipid-based systems (e.g., liposomes) are commonly used in human clinical trials especially in anti-cancer gene therapy [10,35]. Cationic lipids are amphiphilic molecules composed of one or two fatty acid side chains (acyl) or alkyl, a linker and a hydrophilic amino group. The hydrophobic part can be cholesterol-derived moieties. In aqueous media, cationic lipids are assembled into a bilayer vesicular-like structure (liposomes). Liposomes/DNA complex is usually termed a lipoplex. Negatively charged DNA will neutralize cationic liposomes resulting in aggregation and continuous fusion with time while DNA being entrapped during this process. Because of poor stability (i.e., continuous aggregation), lipoplexes are usually administered directly after their formation. The favorable, stable and small lipoplex particles were produced with the development of the novel liposomal formulation, liposomes/protamine/DNA (LPD). Protamine is arginine-rich peptide, which can condense negatively charged DNA before being complexed with cationic lipids [43,54]. Figure 3A shows the lipoplex-mediated transfection. However, one of the most important drawbacks of these systems is the lack of targeting and non-specific interaction with cells [10,35]. Currently, liposomal nanoparticles (LNs) encapsulating therapeutic agents, or liposomal nanomedicines, represent an advanced class of drug delivery systems, with several formulations in clinical trials. Over the past 20 years, a variety of techniques have been developed for encapsulating both conventional drugs (such as anticancer drugs and antibiotics) and the new genetic drugs (plasmid DNA containing therapeutic genes, antisense oligonucleotides and small interfering RNA) within LNs. If the LNs possess certain properties, they tend to accumulate at sites of disease, such as tumors, where the endothelial layer is 'leaky' and allows extravasation of particles with small diameters. These properties include a diameter centered on 100 nm, a high drug-to-lipid ratio, excellent retention of the encapsulated drug, and a long circulation lifetime (> 6 h). These properties permit the LNs to protect their contents during circulation, prevent contact with healthy tissues, and accumulate at sites of disease. Liposomal nanomedicines have the potential to offer new treatments in such areas as cancer therapy, vaccine development and cholesterol management [55].

General overview of different lipid-based particulate delivery systems, their composition, preparation methods, typical size, route of administration and model antigens has been listed by Myschik J. *et al.*, 2009 [56]. Stimuvax (BLP25 liposome vaccine, L-BLP25, Oncothyreon partnered with Merck KGaA) is a cancer vaccine designed to induce an immune response against the extracellular core peptide of MUC1, a type I membrane glycoprotein widely expressed on many tumors (i.e., lung cancer, breast cancer, prostate cancer and colorectal cancer) [57]. Stimuvax consists of MUC1 lipopeptide BLP25 [STAPPAHGVTSAPDTRPAPGSTAPPK (Pal) G], an immunoadjuvant monophosphoryl lipid A, and three lipids (cholesterol, dimyristoyl phosphatidylglycerol, and dipalmitoyl phosphatidylcholine), capable of enhancing the delivery of the vaccine to APCs. A randomized phase II B clinical trial evaluated the effect of Stimuvax on survival and toxicity in 171 patients with stage III B and IV non-small cell lung cancer (NSCLC), after stable disease or response to first-line chemotherapy. Based on these data, Merck is currently conducting three large phase III clinical trials of Stimuvax. This study will involve more than 1300 patients [57].

A cationic lipid DNA complex (CLDC) consisting of DOTIM/cholesterol liposomes and plasmid DNA, containing immunostimulatory CpG and non-CpG motifs has been designed, with potential immunostimulating and anti-neoplastic activities. Upon systemic administration, TLR-directed cationic lipid-DNA complex JVRS-100 enters dendritic cells (DCs) and macrophages; immunostimulatory DNA binds to and activates Toll-like receptors (TLRs), which may result in the generation of anti-tumor natural killer (NK) cell and T-cell responses by the innate immune system. In addition, as a vaccine adjuvant, this agent may induce a strong cytotoxic T-lymphocyte (CTL) response to co-administered antigen. The efficacy of JVRS-100 has been evaluated in phase I clinical trials for the treatment of patients with Relapsed or Refractory Leukemia [ID: NCT00860522].

##### 2.2. Polysaccharides and cationic polymers

Polysaccharides and other cationic polymers have been recently used in pharmaceutical research and industry for their properties to control the release of antibiotics, DNA, proteins, peptides, drugs or vaccines [58]. They have been also extensively studied as non-viral DNA carriers for gene therapy. Different systems were developed in the last years including poly-lysine and its conjugates, diethylaminoethyl-dextran (DEAE-dextran), dextran-spermine polycations, polyethyleneimine (PEI), polyamidoamine dendrimers, lipopolyamines and chitosan [58]. Many other cationic polymers such as chitosans (a biodegradable linear aminopolysaccharides) and dendrimers (highly branched polyamidoamine) were tested for gene transfer [43]. Chitosan is a biodegradable polysaccharide obtained from deacetylated chitin and the commercial product has an average molecular weight ranging between 4 and 20 kDa. It contains several amino groups that in acidic pH may undergo protonation leading to its solubilization in water. Chitosan may also establish electrostatic interactions with the negatively charged DNA to form complexes (polyplexes). Recently, the preparation of chitosan and chitosan/DNA nanospheres has been reported using a novel and simple osmosis-based method [58].

Cationic polymers can be combined with DNA to form a particulate complex, polyplex, capable of gene transfer into the targeted cells. Since they are synthetic compounds, many modifications such as molecular weight and ligand attachment can be easily achieved. The most widely studied polymers for gene therapy include poly (L-lysine) (PLL) and polyethylenimine (PEI). The nature of PEI polymers enables the targeting ligands and/or polyethylene glycol (PEG) (producing sterically stabilized gene carriers) to their surfaces [43]. For example, pegylated PEI polyplexes were linked to tumor specific ligand transferring an asialoglycoprotein and then applied intravenously, resulting in five-fold increase in the transfection efficiency with lower toxicity in comparison with pegylated (transferrin-free) PEI polyplexes [59]. Furthermore, the synthesis of amphiphilic PLL, by linking both PEG and palmitoyl groups to the polymer, reduced toxicity without compromising the gene delivery efficiency [60].

Polymeric vectors prevent immune reactions, minimize spread to non-target tissues and inhibit degradation of DNA by acting as reservoirs [48]. The effect of cationic polyelectrolytes on tumor cells was studied in culture and in mice with transplanted Ehrlich carcinoma or murine leukemia L5178Y in ascites form. Treated mice were given s.c. or i.p. injections of different polycations at non-toxic doses [61]. Survival of solid Ehrlich carcinoma-bearing mice was significantly increased by high-molecular-weight polyethyleneimine, polyvinylamine, and polypropyleneimine at neutral pH administered as late as 5 days after tumor transplant, accompanied by a 10 to 40% reduction in growth of the solid tumor [61]. Survival of mice bearing leukemia cells in ascites form was improved only by polypropyleneimine. Increase in survival also resulted if the polycation was administered up to 9 days before Ehrlich tumor transplant, with no evidence of weight loss in the host at time of tumor transplant. Thus, cationic polymers may be effective by non-specific stimulation of host immune response to transplanted cells as well as by direct electrostatic cytotoxic interaction with tumor cells [61].

##### 2.3. Micro-/Nano-particles

Another approach to DNA-vaccine delivery involves microparticle-based technologies to target APCs [10]. Microencapsulation of DNA, or association of DNA with microcapsules, has led to enhancement of CTL responses to encoded proteins [11].

Biodegradable, non-antigenic poly-lactide polyglycolide (PLGA or PLG) microspheres offer many advantages as a vaccine delivery system. Both cellular and humoral immune responses can be elicited to antigens encapsulated in, or conjugated onto PLG microspheres. Particles used typically range in size from 1 to 10 μm in diameter, a size that is readily phagocytosed by dendritic cells and other antigen-presenting cells (APCs). Microspheres elicit both CD8+ and CD4+ T cell responses by releasing antigen intracellularly [11]. Biodegradable PLGA nanoparticles (NPs) have been investigated for sustained and targeted/localized delivery of different agents, including drugs, proteins and peptides and recently, plasmid DNA owing to their ability to protect DNA from degradation in endolysosomes. PLGA-based nanotechnology has been widely used in diagnosis and treatment of cancer. These NPs have been shown to stimulate the immune response as measured by an increase in IL-2 and IFN-γ in spleen homogenates [62].

The PLGA polymers can offer long-term release of their contents in a pulsatile manner. In the past, their utilization primarily focused on replacement with the multiple immune boosting administrations typically required to induce protective immunity. As a controlled delivery system, PLGA polymers can potentially deliver antigens or adjuvants to a desired location at predetermined rates and durations, effectively regulating the immune response over a period of time. As a vehicle for targeted antigen delivery, PLGA polymers have been reported to effectively aid in directing antigens to APCs by efficiently trafficking through local lymphoid tissue for uptake by DCs. The majority of the existing literature involving PLGA polymers has tended to be focused on PLGA microspheres. In the last 10 years, microspheres have been used extensively for the injectable delivery of vaccine antigens, both for viral and bacterial antigens [62].

Similar to microspheres, PLGA NPs have been shown to effectively enhance immune responses. The major obstacle is providing delivery vehicles with the adequate surface molecules for recognition by the immune system and for more-effective targeting. It is likely, therefore, that future studies of PLGA NPs as vaccine candidates will focus on improving these features, as recently tested by grafting RGD peptides (arginine-glycine-aspartic acid-containing synthetic peptides) covalently onto PEG moieties on the surface of PLGA NPs [62]. These polymers have been designated as feasible candidates for drug delivery systems, anti-cancer agents and vaccine immunotherapy. For example, DNA vaccine delivery to APCs has been facilitated by microencapsulation of plasmid DNA, which encodes HPV E6/E7 antigenic proteins. The capsule is formed from polymeric PGLA microparticles. These resulting microparticles have a greater propensity toward APC uptake compared to naked DNA. This technique allows HPV DNA plasmid to be condensed inside the microparticle. The physical and chemical properties of the PGLA scaffold render DNA inaccessible to nuclease and preventing degradation, allowing for a sustained release of DNA and enhancing transfection efficiency *in vitro *[18]. In mice, microspheres containing HPV plasmid encoding HPV E6/E7 antigens have been shown to elicit a strong antigen-specific cytotoxic T cell response. Using this technology, microencapsulated DNA vaccine termed ZYC-101 encoding multiple HLA-A2 restricted HPV E7 epitopes has undergone Phase I trials in patients with CIN2/3 lesions and high-grade anal intraepithelial neoplasia. In both trials, intramuscularly administered vaccine was well tolerated, and in some patients had resulted in histological regression of the lesions as well as generation of E7-specific IFN-γ expressing T cells. A newer version of the DNA vaccine, ZYC-101a, which encodes HPV16 and HPV18 E6- and E7-derived epitopes has been used in phase II clinical trial in patients with CIN 2/3 lesions [18].

The administration of DNA in a dry-powder formulation of microscopic particles into the skin by a needle-free mechanism is an alternative method for vaccine delivery. Previous *in vivo *studies in mice suggest that particle-mediated epidermal delivery can suppress tumor growth. The studies of phase I clinical trial are currently underway, evaluating the safety and efficacy of particle-mediated epidermal delivery of cancer vaccines in patients with melanoma and in tumors known to express NY-ESO-1 or LAGE-1 with a NY-ESO-1 plasmid DNA cancer vaccine [1].

The multi-functional nano-devices based on the dendritic polymer or dendrimers can also being applied to a variety of cancer therapies to improve their safety and efficacy. Technical advances have been focused on the development of a linking strategy that allows the dendrimer molecules to be linked via complementary oligonucleotides [63]. At present, further applications of dendrimers in photodynamic therapy, boron neutron capture therapy, and gene therapy for cancer are being examined [63].

Recently, the modified fluorescent nanoparticles have been synthesized as a targeting and delivery system, by conjugating both tumor targeting agent and chemokines to the nanoparticles, in order to attract immune cells toward tumor cells. Biodegradable chitosan nanoparticles encapsulating quantum dots were prepared, with suitable surface modification to immobilize both tumor targeting agent and chemokine on their surfaces [64]. Fluorescent chitosan coated quantum dots (QDs) were used to act as bi-functional bridging units between cancer and immune cells. This nanoparticulate form of delivery promises the advantages of enhanced tumor selectivity and longer half-lives, thereby enhancing effectiveness of the immune response and reduction in systemic toxicity [64].

Several recent US and World patents of developing and modifying nanoparticles for the detection, analysis and treatment of cancer have been mentioned. Many applications in vaccine therapy or gene therapy are listed as following:

1. Gene therapy: a) Nanoparticles formed from self-assembled aggregates of amphipathic molecules covalently linked to LM609 antibody and complexed with the plasmid; b) Nanoparticle containing compacted vector formed by successive additions of oppositely charged polyelectrolytes including an incorporation of ligands into the DNA-polyelectrolyte shells which were mixed with Pluronic F127 gel and polyethylenimine [65].

2. Vaccine therapy: a) Nanoparticles/liposomes containing epidermal growth factor receptor vaccine such as the mannan-modified nanoparticle, including mannan-modified recombinant adenoviral EGFR vaccine and protein vaccine, mannan-modified liposome recombinant EGFR gene and protein vaccine; b) Nano vaccines prepared by envelopment through a magnetic ultrasonic process of an MG7-Ag analog epitope polypeptide and CpG ODN from a biological nano-emulsion, a gastric cancer antigen MG7 and a CpG sequence motif containing oligonucleotides serving as an immune adjuvant; c) Nano vaccines/liposomes utilizing MAGE-1 and HSP70 combined to form a fusion gene. The fusion protein and super-antigen Staphylococcal enterotoxin A were combined to form a complex antigenic compound and encapsulated by a nanoliposome [65].

##### 2.4. Cationic peptides/Cell-penetrating peptides (CPP)

Various natural and/or synthetic cell-penetrating peptides (CPP) have known as efficient tools in vaccine design as they are capable of delivering therapeutic targets into cellular compartments. In fact, the cell membrane is impermeable to hydrophilic substances and delivery into cells could be facilitated by linking to CPP. Different cargos such as drugs, peptide/protein, oligonucleotide/DNA/RNA, nanoparticles, liposomes, bacteriophages, fluorescent dyes and quantum dots have been linked to CPPs for intracellular delivery with possible use in future vaccine design [66]. Two applications of CPP already validated in vaccine studies are delivery of tumor-associated antigens into antigen-presenting cells (APCs) and use as a non-viral gene delivery vehicle in DNA vaccines [66]. There are two methods for designing CPP incorporating immunogenic antigens: 1) chemical linking via covalent bonds 2) coupling via recombinant fusion constructs produced by bacterial expression vectors. The orientation of the peptide and cargo and the type of linkage are likely important [66]. In addition, the utilized CPP, attached cargo, concentration and cell type, all significantly affect the mechanism of internalization. The mechanism of cellular uptake and subsequent processing still remains controversial. It is now apparent that CPP mediate intracellular delivery via both endocytic and non-endocytic pathways [66-68]. An attractive feature of using polypeptides as gene delivery vectors is incorporating multiple functional domains into one polypeptide chain, such as a DNA-binding domain linked with a receptor-targeting domain. This kind of polypeptides will recognize and bind to cell surface receptors that are unique to target cells and deliver the bound DNA into the cells through receptor-mediated endocytosis. Therefore, this process may ensure the therapeutic effect in desired cells and limit the potential side effects caused by transgene expression in non-target cells [69]. Figure 3B demonstrates the peptide-based nucleic acid delivery systems.

Oligo-deoxynucleotides (ODN) with immune-stimulating sequences (ISS) containing CpG motifs facilitate the priming of MHC class I- restricted CD8+ T cell responses to proteins or peptides. Therefore, ODN/cationic peptide complexes are potent tools for priming CD8+ T cell immunity [70]. The complex formation required electrostatic linkage of the positively charged peptide to the negatively charged ODN. Conjugation of immunostimulatory DNA or ODN to protein antigens facilitates the rapid, long-lasting, and potent induction of cell-mediated immunity [70]. It was shown that ODN (with or without CpG-containing sequences) are potent Th1-promoting adjuvants when bound to cationic peptides covalently linked to antigenic epitopes, a mode of antigen delivery existing in many viral nucleocapsids [70].

The HIV Tat derived peptide is a small basic peptide that has been successfully shown to deliver a large variety of cargoes, from small particles to proteins, peptides and nucleic acids. The "transduction domain" or region conveying the cell penetrating properties is clearly confined to a small stretch of basic amino acids, with the sequence RKKRRQRRR (residues 49-57) [71,72]. This polycationic nanopeptide is known to be a transfection enhancer of plasmid DNA. The conditions of DNA-peptide complex formation and DNA/Tat ratio have significant impact on the level of transgene expression and degree of DNA protection from nuclease attack [49]. The conjugation of this peptide to ovalbumin (OVA) resulted in efficient stimulation of MHC class I-restricted T cell responses *in vitro *and, more importantly, the generation of CTLs *in vivo *[73]. Also, soluble Tat-antigen conjugates can deliver the antigen directly to the MHC class I processing pathway and thereby increase the generation of antigen-specific CD8+ T cells *in vitro *[73,74]. A fusion protein containing the carboxy-terminal end of Tat (amino acids: 49-86) linked to the HPV16 E7 oncoprotein enhanced tumor specific immune responses *in vivo *[75]. In C57BL/6 mice, E7-Tat mixed with Quil A generated efficient prophylactic and therapeutic suppression of HPV16-positive C3 tumor outgrowth. This study offers a new strategy for improving subunit cancer vaccines [75]. Particularly, a Tat-derived peptide in combination with a PEG-PEI copolymer could be a promising candidate as gene delivery vehicle intended for pulmonary administration. Tat-PEG-PEI represents a new approach to non-viral gene carrier for lung therapy, comprising protection for plasmid DNA, low toxicity and significantly enhanced transfection efficiency under *in vivo *conditions [76].

It has been shown that covalent attachment of low molecular weight polyethyleneimine (PEI) improves Tat peptide mediated gene delivery *in vitro *[77-79]. In our recent study, two delivery systems including polymer PEI 25 kDa and polymer peptide hybrid as PEI600-Tat conjugate were used to compare their efficiency for HPV16 E7 DNA transfection *in vitro*. Our data indicated that both delivery systems including PEI 25 kDa and PEI600-Tat conjugate are efficient tools for E7 gene transfection. In fact, PEI potency for E7 gene transfection is higher than PEI600-Tat *in vitro*, but its toxicity is obstacle *in vivo *[80]. Using HPV16 E7 as a model antigen, the effect of PEI600-Tat conjugate has been evaluated on the potency of antigen-specific immunity in mice model. Assessment of lymphoproliferative and cytokine responses against recombinant E7 protein (rE7) showed that PEI600-Tat/E7DNA complex at certain ratio induces Th1 response. This study has demonstrated that PEI600-Tat conjugate is efficient to improve immune responses *in vivo *[81]. Synthetic peptides containing a nuclear localization signal (NLS) can be bound to the DNA and the resulting DNA-NLS complexes can be recognized as a nuclear import substrate by specific intracellular receptor proteins [8]. For example, conjugation of an NLS to a Minimalistic Immunogenically Defined Gene Expression (MIDGE) vector encoding a truncated and secreted form of BHV-1 glycoprotein D (tgD) improved the tgD expression *in vitro *and induced both humoral and cellular immune responses in mice [8]. This strategy could be applied as an efficient pathway in enhancement of DNA vaccine potency against cancer.

One of the CPPs that have currently received extensive attention in the field of DNA vaccination is the herpes simplex virus (HSV-1) protein VP22 [66]. VP22 can form compacted complexes with short oligonucleotides and form particles of spherical nature with a size range of 0.3 to 1 μm in diameter. These particles entered cells efficiently within 2 to 4 hours. Furthermore, VP22 enables spreading of the antigenic peptide to the cells surrounding the transfected cells [66]. Efforts have been made to increase the potency of DNA vaccines by exploiting the cell-to-cell spreading capabilities of the HSV-1 VP22 protein or the analogous protein from bovin herpesvirus 1 [10]. The significance of VP22 in intercellular spreading has been demonstrated through *in vitro *studies linking VP22 to p53, thymidine kinase, cytosine deaminase and Green Fluorescent Protein (GFP). These proteins were observed to be distributed to nuclei of surrounding cells [18]. Furthermore, vaccination with DNA encoding HPV16E7 linked to the HSV type 1 VP22 elicited the enhanced E7-specific memory CD8+ T lymphocytes and anti-tumor effects against E7-expressing tumor cells [82]. Also, VP22 has been used for HPV DNA vaccines targeting the E6 protein [18]. Various groups have demonstrated that DNA constructs which encode fusion proteins of VP22 linked to an antigen increase the immune responses in mice and cattle. Bovine herpesvirus VP22 (BVP22) and Marek's disease virus VP22 (MVP-1) are both closely related by their structural homology to HSV-1 VP22, and can also have a significant role in intercellular spreading. Hung *et al. *has demonstrated that mice vaccinated with DNA encoding MVP22/E7 significantly increased numbers of IFN-γ-secreting, E7-specific CD8+ T cell precursors compared to mice vaccinated with wild-type E7 DNA alone, which directly lead to a stronger tumor prevention response. Similarly, immunization of mice and cattle with DNA vaccine coding for BVP22 linked to truncated glycoprotein D (BVP-tgD) was shown to generate a stronger tgD-specific immune response compared to animals vaccinated with tgD alone. Taken together, DNA vaccine encoding VP22 linked to antigens represents a promising approach to enhance DNA vaccine potency [18].

However, the data concerning the mechanism responsible for increasing of immune responses are controversial [10]. To evaluate the VP22 role in gene therapy of hepatocellular carcinomas (HCCs), the expression vectors were constructed for N- and C-terminal fragments of VP22-p53 fusion proteins and investigated the VP22-mediated shuttle effect in hepatoma cells by co-transfection experiments. VP22-mediated trafficking was not detectable in hepatoma cells *in vitro *by fluorescence microscopy [83]. For *in vivo *experiments, the recombinant adenoviruses Ad5CMVp53 and Ad5CMVp53-VP22 were constructed. In contrast to the *in vitro *experiments, intercellular trafficking of VP22-p53 could be observed in subcutaneous tumors of hepatoma cells by fluorescence microscopy, indicating a stronger shuttle effect in solid tumors compared to cell culture experiments [83].

### VLPs as an efficient delivery system

Virus-like particles (VLPs) have gained increasing interest for their use as vaccines due to their repetitive antigenic structure that is capable of efficiently activating the immune system. In addition to the use of VLPs as direct immunogens, the efficiency that they stimulate cellular and humoral responses has made them prime candidates as carrier molecules for the delivery of epitopes, DNA and small molecules targeting other diseases [84]. The reason that many VLPs make excellent carrier molecules for the delivery of epitopes in vaccines is most likely because the particulate VLP structure is readily taken up into antigen presenting cells and thus is able to prime long lasting CTL responses as well as antibody responses [84]. Notable work has been done in this area with the hepatitis B core particles, human papillomavirus VLPs and parvovirus VLPs displaying T-cell specific epitopes from another protein on their capsid. These studies demonstrate that like bacterial epitope display systems, VLPs are efficient stimulators of MHC class I and class II responses [84]. Consequently, VLPs have great potential as epitope display systems for other diseases.

Human papillomavirus-like particles (HPV VLP) are a candidate vaccine for prevention of HPV infection and also an immunogenic delivery system for incorporated antigen. For example, an L1-E7 fusion protein has been shown to self-assemble into chimeric VLPs (cVLPs) that can be used to enhance E7-specific cellular immune responses in mice [85]. Also, L2-E7 or L2-E7-E2 fusion proteins have been generated and incorporated into chimeric VLPs that have been shown to provide similar enhancement of E7-and/or E2-specific responses [86,87]. In addition to using VLPs for delivery of viral early proteins, VLPs consisting of L1 alone have been indicated to be capable of delivering plasmid DNA into cells grown *in vitro *[88]. The researchers have shown previously that polyomavirus VP1 VLPs [89,90] or HPVL1 VLPs [91,92], are able to mediate delivery and expression of plasmid DNA *in vitro*. Interestingly, the recent evidence has suggested that VLPs consisting of both the L1 major and L2 minor capsid proteins are more efficient for DNA delivery than VLPs consisting of L1 alone [93]. Kamper *et al. *[94] showed that DNA co-delivered with L1 VLPs is retained within endosomes, and that efficient egress from this compartment is dependent on a 23 amino acid sequence located within the L2 carboxyl-terminal region. Thus, a potentially important role for L2 has been identified in facilitating DNA delivery and expression *in vitro*. These findings support the development of VLP-based strategies for both prophylaxis and therapy of HPV-associated diseases, and for using VLPs in an effort to avoid barriers commonly encountered with DNA-based immunization strategies [88,93]. Additional evidence to support this concept was generated in experiments in which co-administration of VLPs with a plasmid designed to express HPV16 E6 oncoprotein was associated with significant enhancement of plasmid-encoded E6-specific cellular immune responses [93]. Consistent with these findings, co-administration of L1/L2 VLPs with pcDNA-CRT/E6 expression plasmid has been associated with significant enhancement of E6-specific cellular immune responses [93].

L2 has also been shown to mediate co-localization of L1 and DNA within the nucleus in promyelocytic leukemia oncogenic domains (POD), known as ND10 [95]. Although, ND10 function is not yet fully characterized, a role in RNA processing has been suggested [96]. ND10 sites are also known to contain RNA polymerase II, and CBP, a transcriptional co-activator, which supports a transcriptional role for these structures [96]. Thus, L2 may facilitate expression of co-delivered DNA not only by mediating endosomal escape, but also by mediating localization of DNA to sites that support transcription.

An optical imaging approach has been designed to directly visualize the trafficking of *simian-human immunodeficiency *(SHIV) VLPs after immunization by common routes of injection. It was shown that VLPs can easily enter the draining lymph nodes with quantitative differences in the number of lymph node involvement depending on the immunization route. Intradermal immunization led to the largest level of lymph node involvement for the longest period of time, which correlated with the strongest humoral and cellular immune responses. Therefore, intradermal immunization showed improved responses and might be a preferable delivery route for viral and cancer immunotherapeutic studies involving VLPs [97].

### Delivery systems in dendritic cell-based vaccines

Dendritic cells (DCs) are potent antigen-presenting cells capable of initiating a primary immune response and possess the ability to activate T cells and stimulate the growth and differentiation of B cells. DCs provide a direct connection between innate and adaptive immune response, and arise from bone marrow precursors that are present in immature forms in peripheral tissues, where they are prepared to capture antigens. DCs migrate from the peripheral tissues to the closest lymph nodes through afferent lymphatic vessels to present the foreign antigens, stimulating T-cell activation and initiating a cellular immune response [15]. In dendritic cell-based cancer immunotherapy, it is important that DCs present peptides derived from tumor-associated antigens on MHC class I, and activate tumor-specific cytotoxic T lymphocytes. However, MHC class I generally present endogenous antigens expressed in the cytosol. Several researchers have developed antigen delivery tools based on the cross presentation theory of exogenous antigens for DCs. In these studies, various types of antigen delivery carriers such as liposomes [98,99], poly-(γ-glutamic acid) nanoparticles [100] and cholesterol pullulan nanoparticles [101], which can deliver antigen into DCs via the endocytosis pathway, have been used. Furthermore, IgG modified liposomes with entrapped antigen have been reported to induce cross presentation of exogenous antigen for DCs on MHC class I molecules [102]. These carriers deliver antigens into DCs via an endocytosis mechanism, likely due to exogenous antigen leaking from the endosome into the cytosol. In other study, DCs pulsed with exogenous antigens by electroporation presented their antigens on MHC class I molecules and resulted in inducing MHC class I-mediated anti-tumor immunity. Although electroporation is commonly utilized to deliver gene such as DNA and RNA into cytosol, Kim *et al. *and Weiss *et al*. applied this system to antigen delivery into DCs [103,104]. Moreover, Suzuki *et al. *investigated the effect of antigen delivery using perfluoropropane gas-entrapping liposomes (Bubble liposomes, BLs) and ultrasound (US) exposure on MHC class I presentation levels in DCs, as well as the feasibility of using this antigen delivery system in DC-based cancer immunotherapy. DCs were treated with ovalbumin (OVA) as a model antigen. Ovalbumin was directly delivered into the cytosol but not via the endocytosis pathway, and OVA-derived peptides were presented on MHC class I [105]. Immunization with DCs treated with OVA, BLs and US exposure efficiently induced OVA-specific CTLs and resulted in the complete rejection of E.G7-OVA tumors [96]. These data indicate that the combination of BLs and US exposure is a promising antigen delivery system in DC-based cancer immunotherapy.

It is known that DCs have an important role in various diseases particularly in cancer and autoimmune disorders. Therefore, targeting nanoparticles (NPs) to DCs provides a promising strategy for developing an efficient protective immune response. A variety of NPs have been designed with different properties to target DCs for diverse applications. Specific antigens encapsulated by NPs have been used as delivery systems to DCs [106]. For example, DCs have been loaded with HIV-1 p24 proteins adsorbed on the surface of surfactant-free anionic polylactic acid nanoparticles (PLA NPs) and humoral and cellular immune responses were analyzed. The specific levels of serum IgG and intestinal IgA were observed as well as specific CD4+ T cell proliferation in the spleen and mesenteric lymph nodes in CBA/J mice vaccinated with p24-NPs DCs [106]. This novel delivery tool can also be effective in cancer immunotherapy. For example, *in vitro *generation of DCs loaded with tumor-associated antigens has been investigated against human glioblastoma multiforme, an aggressive primary brain tumor [106].

In a study, NPs were not used only to load DCs with the antigen but instead to regulate the antigen release into the DCs and to develop a controlled response. It has been reported that the injection of exosomes derived from DCs loaded with tumor peptides induces a potent anti-tumor immune response with a final eradication of established tumors. Herein, DCs were pulsed with synthetic peptides that represent cytotoxic T-lymphocyte epitopes of HPV16 E7. Other clinical studies in phase I-II were being carried out for a DC vaccine pulsed with multiple peptides for recurrent malignant gliomas. The objective was to determine the safety and induction of the immune response using these vaccinations. Therefore, NPs can contribute to a better design of medical applications by a controlled release of a specific agent with more efficient and specific targeting, affording the opportunity to track them for obtaining information about their bio-distribution at the same time [106].

### Delivery systems in protein/peptide vaccination

Soluble protein-conjugated polysaccharides are poorly immunogenic and require adjuvants, delivery systems or live vectors to boost immune responses following immunization [38]. For optimal performance, antigen delivery vehicles should closely mimic the composition and immunological processing of actual pathogens; they should actively or passively target APCs such as DCs; protect the antigenic protein from degradation; direct the nature of the resulting immune response (i.e., cellular versus humoral responses) and last, induce APC maturation by interacting with elements of the innate immune system such as Toll-like receptors (TLRs). Several strategies have been reported including directly conjugating TLR ligands to protein antigens or co-encapsulating immunostimulatory agents and proteins in liposomes or hydrophobic polymeric particles [107]. Furthermore, an antigen delivery system has been generated which is based on acid-degradable, acetal-cross-linked, hydrogel particles designed for uptake by APCs. Compared to non-degradable systems, these microparticles greatly enhance the efficacy of MHCI antigen presentation and the subsequent activation of CD8+ cytotoxic T lymphocytes (CTLs), which are crucial in cancer immunotherapy [107]. To affect APC maturation, a method has recently reported for the incorporation of an immunostimulatory CpG oligonucleotide into the polymer backbone of the particles. Following phagocytosis by APCs, these particles were designed to degrade in the acidic environment of endosomal vesicles and release their protein as well as a CpG-polymer conjugate capable of binding TLR9, an endosomal receptor for un-methylated viral and bacterial DNA. TLR9 ligation resulted in APC activation and maturation and led to the subsequent migration of APCs to draining lymph nodes [104]. Although, these microparticles were effective in generating antigen specific immunity, they required a relatively high CpG content, which was due to a loss in activity of the CpG caused by its covalent linkage to the polymer scaffold [107].

However, protein delivery is a safe vaccine approach, particularly suitable for inducing immunity against oncoproteins. The HIV-1 Tat protein is capable of delivering biologically-active proteins to the cytoplasmic compartment via the plasma membrane and is independent of cell type [108-111].

Synthetic peptides with the minimal sequences are necessary for immuno-modulation and have attracted considerable attention as a basis for subunit vaccine design. Peptide vaccine efficacy is determined by how the peptides are recognized and processed by the immune system. Specifically, peptide concentration, multi-valency, secondary structure, length and the presence of helper T-cell epitopes can significantly affect the immune response [112]. Conserved microbial motifs can trigger innate responses, through binding to Toll-like receptors (TLRs) on the surface of APCs. Linking peptide antigens with TLR agonists in a single construct has proven to be an effective approach for enhancing peptide immunogenicity. Many designs for delivering antigenic peptides and adjuvants have been explored, including direct peptide-adjuvant conjugates, and particulate systems such as liposomes, virus-like particles, degradable polymers and non-degradable solid-core beads. These delivery vehicles can not only couple peptide antigens with TLR agonists, but also can have immune-stimulating properties, such as DC targeting, multivalent peptide display and additional adjuvant activity and can provide protection against degradation.

The route of administration and the specific vaccine formulation will have a profound effect on factors such as peptide orientation and structure, stability of peptides against degradation and clearance, tissue localization, toxicity and antigen uptake and processing [112].

### Mucosal delivery systems

Prophylactic and therapeutic responses against infectious diseases and cancer can be induced systemically and at mucosal surfaces by activating the mucosal immune system. Different challenges are associated with different types of mucosal vaccines. Thus, administration routes, carrier systems and adjuvants were considered that can be used to overcome these challenges to enhance mucosal vaccination. The use of particle-mediated delivery systems is an effective strategy to enhance mucosal vaccination by protecting immunogenic material during delivery, providing targeted delivery systems, and allowing incorporation of adjuvant material [113]. The mucosal immune system has been established as an ideal target site for vaccines. Many pathogens infect the host at a specific entry site in the mucosal surface, specifically the M-cells. Hence, it can be an effective strategy for vaccination to target the immunization to these cells. Traditionally, vaccines are administered by injection (e.g., intramuscular vaccination) and will most probably elicit systemic immune responses but only insufficient mucosal responses. On the other side, oral or respiratory immunization usually favors the development of mucosal antibodies and cell-mediated immune responses [113]. The efficacy of M-cell delivery of DNA or any other orally administered vaccine is dependent on 1) whether the administered agents can survive into the gastric and intestinal environments, including pH-induced degradation, enzymes, and diffusion across mucus layer; and 2) whether residence time in the intestine is long enough for sufficient interaction with target cells so that these can endocytose the vaccines. Because of this, oral administration of a vaccine often requires delivery systems that can provide protection against enzymatic degradation and elimination in the gastrointestinal tract in order to maintain a high bioavailability [113]. One way to ensure efficacy of immunization is by shielding the payload from the gastrointestinal tract by encapsulation or inclusion into microspheres or a multi-phase systems such as water-oil-water multiple emulsions. Also, biologically active polymers can be used to further broaden the application. Another way to improve vaccine delivery is by extending the intestinal residence time using specific muco- or bio-adhesins binding to intestinal mucus or to the apical surfaces of epithelial cells in the intestine. However, strategic selection of materials used in the development of mucosal vaccine delivery can also benefit from intrinsic immunoadjuvants effect (e.g., Pluronic copolymers and squalane oil) to enhance the immunogenic response and improve the vaccine efficacy [107]. Successful mucosal vaccination for acute and chronic diseases will require greater understanding of disease pathology, advances in the biology of mucosal immunity in the nasal and gastrointestinal tract, development of multivalent antigens that can elicit potent and long-lasting immune responses and advances in materials science and technology that can be used to develop more effective and targeted delivery systems [113].

### Non-invasive techniques for monitoring *in vivo *antigen capture and delivery

A major parameter limiting immune responses to vaccination is the number of activated APCs that capture antigen and migrate to draining lymph nodes. The use of cellular magnetic resonance imaging (MRI) is a promising approach for this purpose [114]. In a study, an *in vivo *labeling method was described, which relies upon cell-to-cell transfer of super-paramagnetic iron oxide (SPIO) from tumor cells to endogenous APCs, *in situ*, for quantification of APC delivery to lymph nodes in a tumor vaccine model. Mice were immunized with a tumor cell-based vaccine that was labeled with SPIO. APCs that had captured SPIO were imaged over time as they accumulated in lymph nodes. It was indicated that MRI is capable of monitoring, *in vivo*, the trafficking of magnetically labeled APCs inducing a tumor-specific immune response, and that these cells can be magnetically recovered *ex vivo*. Excellent correlation was observed between *in vivo *and *ex vivo *quantification of APCs, with resolution sufficient to detect increased APC trafficking elicited by an adjuvant [114].

Furthermore, the rapid development of Quantum Dots (QDs) technology has already fulfilled some of the hopes of developing new, more effective cancer-imaging probes. First, stable encapsulation of QDs with amphiphilic polymers has prevented the quenching of QD fluorescence in the aqueous *in vivo *environment. Second, QDs are relatively inert and stable. Finally, successful conjugation of QDs with biomolecules has probably made active targeting them to tumors. Despite their success so far in cancer imaging, there are challenges in enhancing sensitivity, maximizing specificity and minimizing toxicity of QDs, which must be undertaken before clinical applications can proceed [115].

## Conclusion

The major aim in gene therapy is to develop efficient, non-toxic gene carriers that can encapsulate and deliver foreign genetic materials into specific cell types including cancerous cells. Both viral and non-viral vectors were developed and evaluated for delivering therapeutic genes into cancer cells. Many viruses such as *retroviru*s, *adenovirus*, *herpes simplex virus*, *adeno-associated virus *and *pox virus *have been modified to eliminate their toxicity and maintain their high gene transfer capability. Due to the limitations correlated to viral vectors, non-viral vectors have been further focused as an alternative in delivery systems. Non-viral vectors include cationic polymers such as polyethylenimine (PEI), polylysine (PLL), cationic peptides and cationic liposomes. Currently, many modifications to the current delivery systems and novel carrier systems have been developed to optimize the transfection efficiency. Furthermore, the route of immunization can influence the outcome of the immune response through altering the interaction between the vaccine and different APCs at the site of injection. Hence, the routes of administration and formulation of DNA clearly affect the therapeutic response by altering immune pathway. Among the commonly used methods of DNA vaccination, the highest efficacy was achieved after *in vivo *electroporation and gene gun delivery. However, it is critical to further analyze the results of ongoing clinical trials, specifically, in the aspect of their success or failure of certain delivery methodologies for DNA vaccines.

## Competing interests

The authors declare that they have no competing interests.

## Authors' contributions

All authors read and approved the final manuscript.

## References

[B1] Bodles-Brakhop AM Draghia-Akli R DNA vaccination and gene therapy: optimization and delivery for cancer therapy Expert Rev Vaccines 2008 7 1085 1101 10.1586/14760584.7.7.1085 18767956

[B2] Bolhassani A Mohit E Rafati S Different spectra of therapeutic vaccine development against HPV infections Human Vaccines 2009 5 671 689 10.4161/hv.5.10.9370 19684468

[B3] Palena C Abrams SI Schlom J Hodge JW Cancer vaccines: Preclinical studies and novel strategies Advances in Cancer Research 2006 115 137 10.1016/S0065-230X(06)95004-0 16860657

[B4] Fioretti D Iurescia S Fazio VM Rinaldi M DNA Vaccines: Developing New Strategies against Cancer Journal of Biomedicine and Biotechnology 2010 1 16 10.1155/2010/174378 PMC2846346 20368780

[B5] Schweighoffer T Molecular cancer vaccines: Tumor therapy using antigen-specific immunizations Pathology & Oncology Research 1997 3 3 164 176 10.1007/BF02899917 18470726

[B6] Hung CF Monie A Alvarez RD Wu TC DNA vaccines for cervical cancer: from bench to bedside Experimental and Molecular Medicine 2007 39 679 689 18160838 10.1038/emm.2007.74 PMC3181139

[B7] Hung CF Monie A Alvarez RD Wu TC DNA vaccines for cervical cancer: from bench to bedside Experimental and Molecular Medicine 2007 39 679 689 18160838 10.1038/emm.2007.74 PMC3181139

[B8] Zheng C Juhls C Oswald D Sack F Westfehling I Wittig B Babiuk LA Hurk SDL Effect of different nuclear localization sequences on the immune responses induced by a MIDGE vector encoding bovine herpesvirus-1 glycoprotein D Vaccine 2006 24 4625 4629 10.1016/j.vaccine.2005.08.046 16154243

[B9] Kutzler MA Weiner DB DNA vaccines: ready for prime time? Nat Rev 2008 9 776 788 10.1038/nrg2432 PMC4317294 18781156

[B10] Ulmer JB Wahren B Liu MA Gene-based vaccines: recent technical and clinical advances Trends in Molecular Medicine 2006 12 216 222 10.1016/j.molmed.2006.03.007 16621717

[B11] Doria-Rose NA Haigwood NL DNA vaccine strategies: candidates for immune modulation and immunization regimens Methods 2003 31 207 216 10.1016/S1046-2023(03)00135-X 14511953

[B12] Mikszta JA Laurent PE Cutaneous delivery of prophylactic and therapeutic vaccines: historical perspective and future outlook Expert Rev Vaccines 2008 7 1329 1339 10.1586/14760584.7.9.1329 18980537

[B13] Zaman M Simerska P Toth I Synthetic polyacrylate polymers as particulate intranasal vaccine delivery systems for the induction of mucosal immune response Curr Drug Deliv 2010 7 2 118 24 10.2174/156720110791011846 20158484

[B14] Lai MD Yen MC Lin CM Tu CF Wang CC Lin PS Yang HJ Lin CC The effects of DNA formulation and administration route on cancer therapeutic efficacy with xenogenic EGFR DNA vaccine in a lung cancer animal model Genetic Vaccines and Therapy 2009 7 1 13 10.1186/1479-0556-7-2 19178753 PMC2645394

[B15] Pokorna D Rubio I Müller M DNA-vaccination via tattooing induces stronger humoral and cellular immune responses than intramuscular delivery supported by molecular adjuvants Genet Vaccines Ther 2008 6 1 8 10.1186/1479-0556-6-4 18257910 PMC2267179

[B16] Pokorna D Polakova I Kindlova M Du skova M Ludvikova V Gabriel P Kutinova L Muller M Smahel M Vaccination with human papillomavirus type 16-derived peptides using a tattoo device Vaccine 2009 27 3519 3529 10.1016/j.vaccine.2009.03.073 19464530

[B17] Aravindaram K Yang NS Gene gun delivery systems for cancer vaccine approaches Methods Mol Biol 2009 542 167 178 full_text 19565902 10.1007/978-1-59745-561-9_9

[B18] Lin K Roosinovich E Ma B Hung CF Wu TC Therapeutic HPV DNA vaccines Immunol Res 2010 1 27 10.1007/s12026-009-8141-6 PMC2891127 20066511

[B19] Liu L Zhou X Liu H Xiang L Yuan Z CpG motif acts as a 'danger signal' and provides a T helper type 1-biased microenvironment for DNA vaccination Immunology 2005 115 223 230 10.1111/j.1365-2567.2005.02150.x 15885128 PMC1782151

[B20] Trimble C Lin CT Hung CF Pai S Juang J He L Gillison M Pardoll D Wu L Wu TC Comparison of the CD8+ T cell responses and antitumor effects generated by DNA vaccine administered through gene gun, biojector and syringe Vaccine 2003 21 4036 4042 10.1016/S0264-410X(03)00275-5 12922140

[B21] Fechheimer M Boylan JF Parker S Sisken JE Patel GL Zimmer SG Transfection of mammalian cells with plasmid DNA by scrape loading and sonication loading Proc Natl Acad Sci USA 1987 84 8463 8467 10.1073/pnas.84.23.8463 2446324 PMC299564

[B22] Miller MW Miller DL Brayman AA A review of in vitro bioeffects of inertial ultrasonic cavitation from a mechanistic perspective Ultrasound Med Biol 1996 22 1131 1154 10.1016/S0301-5629(96)00089-0 9123638

[B23] Shen ZP Brayman AA Chen L Miao CH Ultrasound with microbubbles enhances gene expression of plasmid DNA in the liver via intraportal delivery Gene Ther 2008 15 1147 1155 10.1038/gt.2008.51 18385766 PMC3747825

[B24] Bekeredjian R Kuecherer HF Kroll RD Katus HA Hardt SE Ultrasound-targeted microbubble destruction augments protein delivery into testes Urology 2007 69 386 389 10.1016/j.urology.2006.12.004 17320694

[B25] Wallace M Evans B Woods S Mogg R Zhang L Finnefrock AC Rabussay D Fons M Mallee J Mehrotra D Schodel F Musey L Tolerability of two sequential electroporation treatments using MedPulser DNA delivery system (DDS) in healthy adults The American Society of Gene Therapy 2009 17 922 928 10.1038/mt.2009.27 PMC2835142 19277016

[B26] Sundararajan R Nano-electroporation: A first look Methods in Molecular Biology 2008 423 109 128 full_text 18370193 10.1007/978-1-59745-194-9_7

[B27] Hu H Huang X Tao L Huang Y Cui B Wang H Comparative analysis of the immunogenicity of SARS-CoV nucleocapsid DNA vaccine administrated with different routes in mouse model Vaccine 2009 27 1758 1763 10.1016/j.vaccine.2009.01.021 19186202 PMC7115532

[B28] Buchan S Grønevik E Mathiesen I King CA Stevenson FK Rice J Electroporation as a "prime/boost" strategy for naked DNA vaccination against a tumor antigen J Immunol 2005 174 6292 6298 15879128 10.4049/jimmunol.174.10.6292

[B29] Ahmad S Casey G Sweeney P Tangney M O'Sullivan GC Optimized electroporation mediated DNA vaccination for treatment of prostate cancer Genetic Vaccines and Therapy 2010 8 1 13 10.1186/1479-0556-8-1 20181099 PMC2829554

[B30] Seo SH Jin HT Park SH Youn JI Sung YC Optimal induction of HPV DNA vaccine-induced CD8+ T cell responses and therapeutic antitumor effect by antigen engineering and electroporation Vaccine 2009 27 5906 5912 10.1016/j.vaccine.2009.07.033 19651174

[B31] Best SR Peng S Juang CM Hung CF Hannaman D Saunders JR Wu TC Pai SI Administration of HPV DNA vaccine via electroporation elicits the strongest CD8+ T cell immune responses compared to intramuscular injection and intradermal gene gun delivery Vaccine 2009 27 5450 5459 10.1016/j.vaccine.2009.07.005 19622402 PMC2745985

[B32] Daud AI DeConti RC Andrews S Urbas P Riker AI Sondak VK Munster PN Sullivan DM Ugen KE Messina JL Heller R Phase I Trial of Interleukin-12 Plasmid Electroporation in Patients With Metastatic Melanoma Journal of Clinical Oncology 2008 26 36 5896 5903 19029422 10.1200/JCO.2007.15.6794 PMC2645111

[B33] Bodles-Brakhop AM Heller R Draghia-Akli R Electroporation for the delivery of DNA-based vaccines and immunotherapeutics: current clinical developments Molecular Therapy 2009 17 4 585 592 10.1038/mt.2009.5 19223870 PMC2835112

[B34] Kraft SD Richter C Zeil K Baumann M Beyreuther E Bock S Bussmann M Cowan TE Dammene Y Enghardt W Helbig U Karsch L Kluge T Laschinsky L Lessmann E Metzkes J Naumburger D Sauerbrey R Schürer M Sobiella M Woithe J Schramm U Pawelke J Dose dependent biological damage of tumor cells by laser-accelerated proton beams New Journal of Physics 2010 12 10.1088/1367-2630/12/8/085003

[B35] Luo D Saltzman WM Synthetic DNA delivery systems Nat Biotechnol 2000 18 33 37 10.1038/71889 10625387

[B36] Azevedo V Levitus G Miyoshi A Cândido AL Goes AM Oliveira SC Main features of DNA-based immunization vectors Braz J Med Biol Res 1999 32 2 147 153 10.1590/S0100-879X1999000200002 10347749

[B37] Hilleman MR Overview of vaccinology with special reference to papillomavirus vaccines J Clin Virol 2000 19 79 90 10.1016/S1386-6532(00)00132-3 11091151

[B38] Mills KHG Designer adjuvants for enhancing the efficacy of infectious disease and cancer vaccines based on suppression of regulatory T cell induction Immunology Letters 2009 122 108 111 10.1016/j.imlet.2008.11.007 19100777

[B39] Marconi P Argnani R Epstein AL Manservigi R HSV as a Vector in Vaccine Development and Gene Therapy Adv Exp Med Biol 2009 655 118 144 full_text 20047039 10.1007/978-1-4419-1132-2_10

[B40] Singh R Kostarelos K Designer adenoviruses for nanomedicine and nanodiagnostics Trends Biotechnol 2009 27 220 9 10.1016/j.tibtech.2009.01.003 19251331

[B41] Basak SK Kiertscher SM Harui A Roth MD Modifying Adenoviral Vectors for Use as Gene-Based Cancer Vaccines Viral Immunology 2004 17 2 182 196 10.1089/0882824041310603 15279698

[B42] Barzon L Bonaguro R Castagliuolo I Chilosi M Franchin E Del Vecchio C Giaretta I Boscaro M Palu G Gene therapy of thyroid cancer via retrovirally-driven combined expression of human IL-2 and herpes simplex virus thymidine kinase Eur J Endocrinol 2003 148 73 80 10.1530/eje.0.1480073 12534360

[B43] El-Aneed A An overview of current delivery systems in cancer gene therapy Journal of Controlled Release 2004 94 1 14 10.1016/j.jconrel.2003.09.013 14684267

[B44] Kaufman HL Flanagan K Lee CS Perretta DJ Horig H Insertion of IL-2 and IL-12 genes into vaccinia virus results in effective anti-tumor responses without toxicity Vaccine 2002 20 1862 1869 10.1016/S0264-410X(02)00032-4 11906776

[B45] Moreno M Kramer MG Yim L Chabalgoity JA *Salmonella *as live trojan horse for vaccine development and cancer gene therapy Curr Gene Ther 2010 10 56 76 10.2174/156652310790945566 20156188

[B46] Kim-Schulze S Kaufman HL Walther W, Stein US Gene therapy for anti-tumor vaccination Methods in Molecular Biology, Gene Therapy of Cancer 542 515 527 full_text 10.1007/978-1-59745-561-9_27 19565920

[B47] Vergati M Intrivici C Huen NY Schlom J Tsang KY Strategies for cancer vaccine development Journal of Biomedicine and Biotechnology 2010 1 13 10.1155/2010/596432 PMC2914453 20706612

[B48] Narayani R Polymeric delivery systems in biotechnology: a mini review Trends Biomater Artif Organs 2007 21 14 19

[B49] Hellgren I Gorman J Sylven C Factors controlling the efficiency of Tat-mediated plasmid DNA transfer J Drug Target 2004 12 39 47 10.1080/106118604200041403 15203910

[B50] Khatri K Goyal AK Vyas SP Potential of nanocarriers in genetic immunization Recent Pat Drug Deliv Formul 2008 2 68 82 10.2174/187221108783331348 19075899

[B51] Martin ME Rice KG Peptide-guided gene delivery AAPS J 2007 9 18 29 10.1208/aapsj0901003 PMC2751301 17408236

[B52] Lee TWR Matthews DA Blair GE Novel molecular approaches to cystic fibrosis gene therapy Biochem J 2005 387 1 15 10.1042/BJ20041053 15656784 PMC1134927

[B53] Narayani R Polymeric delivery systems in biotechnology: A mini-Review Trends Biomater Artif Organs 2007 21 14 19

[B54] Gao X Huang L Potentiation of cationic liposome-mediated gene delivery by polycations Biochemistry 1996 35 1027 1036 10.1021/bi952436a 8547238

[B55] Fenske DB Cullis PR Liposomal nanomedicines Expert Opin Drug Deliv 2008 5 25 44 10.1517/17425247.5.1.25 18095927

[B56] Myschik J Rades T Hook S Advances in lipid-based subunit vaccine formulations Current Immunology Reviews 2009 5 42 48 10.2174/157339509787314378

[B57] Vergati M Intrivici C Huen NY Schlom J Tsang KY Strategies for cancer vaccine development Journal of Biomedicine and Biotechnology 2010 1 13 10.1155/2010/596432 PMC2914453 20706612

[B58] Masotti A Ortaggi G Chitosan micro- and nanospheres: fabrication and applications for drug and DNA delivery Mini Rev Med Chem 2009 9 463 469 10.2174/138955709787847976 19356124

[B59] Kircheis R Schuller S Brunner S Ogris M Heider KH Zauner W Wagner E Polycation-based DNA complexes for tumor-targeted gene delivery *in vivo* J Gene Med 1999 1 111 120 10.1002/(SICI)1521-2254(199903/04)1:2<111::AID-JGM22>3.0.CO;2-Y 10738575

[B60] Brown MD Schatzlein A Brownlie A Jack V Wang W Tetley L Gray AI Uchegbu IF Preliminary characterization of novel amino acid based polymeric vesicles as gene and drug delivery agents Bioconjug Chem 2000 11 880 891 10.1021/bc000052d 11087338

[B61] Moroson H Polycation-treated tumor cells *in vivo *and *in vitro* Cancer Research 1971 31 373 380 4100677

[B62] Lu JM Wang X Marin-Muller C Wang H Lin PH Yao Q Chen C Current advances in research and clinical applications of PLGA-based nanotechnology Expert Rev Mol Diagn 2009 9 325 341 10.1586/erm.09.15 19435455 PMC2701163

[B63] Baker JR Jr Dendrimer-based nanoparticles for cancer therapy Nanotechnology for Hematology 2009 708 719 10.1182/asheducation-2009.1.708 20008257

[B64] Chatterjee DK Zhang Y Multi-functional nanoparticles for cancer therapy Science and Technology of Advanced Materials 2007 8 131 133 10.1016/j.stam.2006.09.008

[B65] Praetorius NP Mandal TK Engineered nanoparticles in cancer therapy Recent Patents on Drug Delivery & Formulation 2007 1 37 51 10.2174/187221107779814104 19075873

[B66] Brooks NA Pouniotis DS Tang CK Apostolopoulos V Pietersz GA Cell-penetrating peptides: application in vaccine delivery Biochimica et biophysica Acta 2010 1805 25 34 19782720 10.1016/j.bbcan.2009.09.004

[B67] Jarver P Langel U The use of cell-penetrating peptides as a tool for gene regulation DDT 2004 9 395 402 15081956 10.1016/S1359-6446(04)03042-9

[B68] Wagstaff KM Jans DA Protein transduction: cell penetrating peptides and their therapeutic applications Current Medicinal Chemistry 2006 13 1371 1387 10.2174/092986706776872871 16719783

[B69] Zeng J Wang S Enhanced gene delivery to PC12 cells by a cationic polypeptide Biomaterials 2005 26 679 686 10.1016/j.biomaterials.2004.03.006 15282146

[B70] Schirmbeck R Riedl P Zurbriggen R Akira S Reimann J Antigenic epitopes fused to cationic peptide bound to oligonucleotides facilitate toll-like receptor 9-dependent, but CD4+ T cell help-independent, priming of CD8+ T cells The Journal of Immunology 2003 171 5198 5207 14607920 10.4049/jimmunol.171.10.5198

[B71] Riedl P Reimann J Schirmbeck R Peptides containing antigenic and cationic domains have enhanced, multivalent immunogenicity when bound to DNA vaccines J Mol Med 2004 82 144 152 10.1007/s00109-003-0502-3 14652667

[B72] Brooks H Lebleu B Vives E Tat peptide-mediated cellular delivery: back to basics Adv Drug Deliv Rev 2005 57 559 577 10.1016/j.addr.2004.12.001 15722164

[B73] Kim DT Mitchell DJ Brockstedt DG Fong L Nolan GP Fathman CG Engleman EG Rothbard JB Introduction of soluble proteins into the MHC class I pathway by conjugation to an HIV tat peptide J Immunol 1997 159 1666 1668 9257826

[B74] Riedl P Reimann J Schirmbeck R Complexes of DNA vaccines with cationic, antigenic peptides are potent, polyvalent CD8+ T cell-stimulating immunogens Methods Mol Med 2004 127 159 169 10.1385/1-59745-168-1:159 16988454

[B75] Giannouli C Brulet JM Gesché F Rappaport J Burny A Leo O Hallez S Fusion of a tumor-associated antigen to HIV-1 Tat improves protein-based immunotherapy of cancer Anticancer Res 2003 23 3523 3532 12926102

[B76] Kleemann E Neu M Jekel N Fink L Schmehl T Gessler T Seeger W Kissel T Nano-carriers for DNA delivery to the lung based upon a TAT-derived peptide covalently coupled to PEG-PEI Journal of Controlled Release 2005 109 299 316 10.1016/j.jconrel.2005.09.036 16298009

[B77] Alexis F Lo SL Wang S Covalent attachment of low molecular weight poly (ethyleneimine) improves Tat peptide mediated gene delivery Adv Mater 2006 18 2174 2178 10.1002/adma.200502173

[B78] Putnam D Gentry CA Pack DW Langer R Polymer based gene delivery with low cytotoxicity by a unique balance of side-chain termini Proc Natl Acad Sci 2001 98 1200 1205 10.1073/pnas.031577698 11158617 PMC14732

[B79] Wang S Tat peptide conjugates of low molecular weight polyethylenimine as effective non-viral gene delivery vectors Mol Ther 2006 13 76 10.1016/j.ymthe.2006.08.219

[B80] Bolhassani A Ghasemi N Servis C Taghikhani M Rafati S Comparison of two delivery systems efficiency by using polyethylenimine (PEI) for plasmid HPV16 E7 DNA transfection into COS-7 cells Modarres J Med Sci 2008 11 15 19

[B81] Bolhassani A Ghasemi N Servis C Taghikhani M Rafati S The efficiency of a novel delivery system (PEI600-Tat) in development of potent DNA vaccine using HPV16 E7 as a model antigen Drug Deliv 2009 16 196 204 10.1080/10717540902757721 19514980

[B82] Michel N Osen W Gissmann L Schumacher TN Zentgraf H Müller M Enhanced immunogenicity of HPV16 E7 fusion proteins in DNA vaccination Virology 2002 294 47 59 10.1006/viro.2001.1321 11886264

[B83] Zender L Kuhnel F Kock R Manns M Kubicka S VP22-mediated intercellular transport of p53 in hepatoma cells *in vitro *and *in vivo* Cancer Gene Therapy 2002 9 489 496 10.1038/sj.cgt.7700465 12032659

[B84] Roy P Noad R Virus-like particles as a vaccine delivery system Human Vaccines 2008 4 5 12 18438104 10.4161/hv.4.1.5559

[B85] Schafer K Muller M Faath S Henn A Osen W Zentgraf H Immune response to human papillomavirus 16L1E7 chimeric virus-like particles: induction of cytotoxic T cells and specific tumor protection Int J Cancer 1999 81 881 888 10.1002/(SICI)1097-0215(19990611)81:6<881::AID-IJC8>3.0.CO;2-T 10362134

[B86] Greenstone HL Nieland JD de Visser KE De Bruijn ML Kirnbauer R Roden RBS Lowy DR Kast WM Schiller JT Chimeric papillomavirus virus-like particles elicitanti-tumor immunity against the E7 oncoprotein in an HPV16 tumor model Proc Natl Acad Sci USA 1998 95 1800 1805 10.1073/pnas.95.4.1800 9465097 PMC19193

[B87] Schiller J Lowy D Papillomavirus-like particle vaccines J Natl Cancer Inst Monogr 2001 28 50 54 11158207 10.1093/oxfordjournals.jncimonographs.a024258

[B88] Kanodia S Fahey LM Kast WM Mechanisms used by human papillomaviruses to escape the host immune response Curr Cancer Drug Targets 2007 7 79 89 10.2174/156800907780006869 17305480

[B89] Krauzewicz N Cox C Soeda E Clark B Rayner S Griffin BE Sustained *ex vivo *and *in vivo *transfer of a reporter gene using polyoma virus pseudocapsids Gene Ther 2000 7 1094 1102 10.1038/sj.gt.3301219 10918475

[B90] Krauzewicz N Stokrova J Jenkins C Elliott M Higgins CF Griffin BE Virus-like gene transfer into cells mediated by polyoma virus pseudocapsids Gene Ther 2000 7 2122 2131 10.1038/sj.gt.3301322 11223994

[B91] Combita AL Touze A Bousarghin L Sizaret PY Munoz N Coursaget P Gene transfer using human papillomavirus pseudovirions varies according to virus genotype and requires cell surface heparan sulfate FEMS Microbiol Lett 2001 204 183 188 10.1111/j.1574-6968.2001.tb10883.x 11682199

[B92] Touze A Coursaget P *In vitro *gene transfer using human papillomavirus-like particles Nucleic Acids Res 1998 26 1317 1323 10.1093/nar/26.5.1317 9469843 PMC147398

[B93] Malboeuf CM Simon DAL Lee YEE Lankes HA Dewhurst S Frelinger JG Rose RC Human papillomavirus-like particles mediate functional delivery of plasmid DNA to antigen presenting cells *in vivo* Vaccine 2007 25 3270 3276 10.1016/j.vaccine.2007.01.067 17293010

[B94] Kamper N Day PM Nowak T Selinka HC Florin L Bolscher J Hilbig L Schiller JT Sapp M A membrane-destabilizing peptide in capsid protein L2 is required for egress of papillomavirus genomes from endosomes J Virol 2006 80 759 768 10.1128/JVI.80.2.759-768.2006 16378978 PMC1346844

[B95] Day PM Baker CC Lowy DR Schiller JT Establishment of papillomavirus infection is enhanced by promyelocytic leukemia protein (PML) expression Proc Natl Acad Sci USA 2004 101 14252 14257 10.1073/pnas.0404229101 15383670 PMC521143

[B96] Kiesslich A von Mikecz A Hemmerich P Cell cycle-dependent association of PML bodies with sites of active transcription in nuclei of mammalian cells J Struct Biol 2002 140 167 79 10.1016/S1047-8477(02)00571-3 12490165

[B97] Cubas R Zhang S Kwon S Sevick-Muraca EM Li M Chen C Yao Q Virus-like particle (VLP) lymphatic trafficking and immune response generation after immunization by different routes J immunother 2009 32 118 128 10.1097/CJI.0b013e31818f13c4 19238010 PMC2717166

[B98] Machy P Serre K Leserman L Class I-restricted presentation of exogenous antigen acquired by Fcgamma receptor-mediated endocytosis is regulated in dendritic cells Eur J Immunol 2000 30 848 857 10.1002/1521-4141(200003)30:3<848::AID-IMMU848>3.0.CO;2-Q 10741401

[B99] Okada N Saito T Mori K Masunaga Y Fujii Y Fujita J Fujimoto K Nakanishi T Tanaka K Nakagawa S Mayumi T Fujita T Yamamoto A Effects of lipofectin-antigen complexes on major histocompatibility complex class I-restricted antigen presentation pathway in murine dendritic cells and on dendritic cell maturation Biochim Biophys Acta 2001 1527 97 101 11479025 10.1016/s0304-4165(01)00160-x

[B100] Yoshikawa T Okada N Oda A Matsuo K Mukai Y Yoshioka Y Akagi T Akashi M Nakagawa S Development of amphiphilic gamma-PGA-nanoparticle based tumor vaccine: potential of the nanoparticulate cytosolic protein delivery carrier Biochem Biophys Res Commun 2008 366 408 413 10.1016/j.bbrc.2007.11.153 18068668

[B101] Wang L Ikeda H Ikuta Y Schmitt M Miyahara Y Takahashi Y Gu X Nagata Y Sasaki Y Akiyoshi K Sunamoto J Nakamura H Kuribayashi K Shiku H Bone marrow-derived dendritic cells incorporate and process hydrophobized poly-saccharide/oncoprotein complex as antigen presenting cells Int J Oncol 1999 14 695 701 10087316 10.3892/ijo.14.4.695

[B102] Kawamura K Kadowaki N Suzuki R Udagawa S Kasaoka S Utoguchi N Kitawaki T Sugimoto N Okada N Maruyama K Uchiyama T Dendritic cells that endocytosed antigen-containing IgG-liposomes elicit effective antitumor immunity J Immunother 2006 29 165 174 10.1097/01.cji.0000190169.61416.f5 16531817

[B103] Kim KW Kim SH Jang JH Lee EY Park SW Um JH Lee YJ Lee CH Yoon S Seo SY Jeong MH Lee ST Chung BS Kang CD Dendritic cells loaded with exogenous antigen by electroporation can enhance MHC class I-mediated antitumor immunity Cancer Immunol Immunother 2004 53 315 322 10.1007/s00262-003-0461-0 14685778 PMC11034209

[B104] Weiss JM Allen C Shivakumar R Feller S Li LH Liu LN Efficient responses in a murine renal tumor model by electroloading dendritic cells with whole-tumor lysate J Immunother 2005 28 542 550 10.1097/01.cji.0000179437.95335.23 16224271

[B105] Suzuki R Oda Y Utoguchi N Namai E Taira Y Okada N Kadowaki N Kodama T Tachibana K Maruyama K A novel strategy utilizing ultrasound for antigen delivery in dendritic cell-based cancer immunotherapy Journal of Controlled Release 2009 133 198 205 10.1016/j.jconrel.2008.10.015 19000727

[B106] Klippstein R Pozo D Nanotechnology-based manipulation of dendritic cells for enhanced immunotherapy strategies Nanomedicine 2010 1 7 10.1016/j.nano.2010.01.001 20085824

[B107] Beaudette TT Bachelder EM Cohen JA Obermeyer AC Broaders KE Fréchet JM Kang ES Mende I Tseng WW Davidson MG Engleman EG *In vivo *studies on the effect of co-encapsulation of CpG DNA and antigen in acid-degradable microparticle vaccines Mol Pharm 2009 6 4 1160 1169 10.1021/mp900038e 19415922 PMC2731711

[B108] Moingeon P Cancer vaccines Vaccine 2001 19 1305 1326 10.1016/S0264-410X(00)00372-8 11163653

[B109] Coulie PG Human tumor antigens recognized by T cells: new perspectives for anti-cancer vaccines? Mol Med Today 1997 3 261 268 10.1016/S1357-4310(97)01049-6 9211417

[B110] Vocero-Akbani A Lissy NA Dowdy SF Transduction of full-length Tat fusion proteins directly into mammalian cells: analysis of T cell receptor activation-induced cell death Methods Enzymol 2000 322 508 521 full_text 10914043 10.1016/s0076-6879(00)22046-6

[B111] Fawell S Seery J Daikh Y Moore C Chen LL Pepinsky B Barsoum J Tat-mediated delivery of heterologous proteins into cells Proc Natl Acad Sci USA 1994 91 664 668 10.1073/pnas.91.2.664 8290579 PMC43009

[B112] Black M Trent A Tirrell M Olive C Advances in the design and delivery of peptide subunit vaccines with a focus on Toll-like receptor agonists Expert Rev Vaccines 2010 9 157 173 10.1586/erv.09.160 20109027 PMC2837080

[B113] Chadwick S Kriegel C Amiji M Delivery strategies to enhance mucosal vaccination Expert Opin Biol Ther 2009 9 427 440 10.1517/14712590902849224 19344280

[B114] Long CM van Laarhoven HWM Bulte JWM Levitsky HI Magnetovaccination as a novel method to assess and quantify dendritic cell tumor antigen capture and delivery to lymph nodes Cancer Res 2009 69 3180 3187 10.1158/0008-5472.CAN-08-3691 19276358 PMC3031988

[B115] Zhang H Yee D Wang C Quantum Dots for cancer diagnosis and therapy: biological and clinical perspectives Nanomedicine 2008 3 83 91 10.2217/17435889.3.1.83 18393668

